# Imaging drug delivery to the lungs: Methods and applications in oncology

**DOI:** 10.1016/j.addr.2022.114641

**Published:** 2023-01

**Authors:** Francis Man, Jie Tang, Magda Swedrowska, Ben Forbes, Rafael T.M. de Rosales

**Affiliations:** a School of Cancer & Pharmaceutical Sciences, King’s College London, London, SE1 9NH, United Kingdom; b School of Biomedical Engineering & Imaging Sciences, King’s College London, London SE1 7EH, United Kingdom

**Keywords:** Drug delivery, Imaging, Lung cancer

## Abstract

Direct delivery to the lung via inhalation is arguably one of the most logical approaches to treat lung cancer using drugs. However, despite significant efforts and investment in this area, this strategy has not progressed in clinical trials. Imaging drug delivery is a powerful tool to understand and develop novel drug delivery strategies. In this review we focus on imaging studies of drug delivery by the inhalation route, to provide a broad overview of the field to date and attempt to better understand the complexities of this route of administration and the significant barriers that it faces, as well as its advantages. We start with a discussion of the specific challenges for drug delivery to the lung via inhalation. We focus on the barriers that have prevented progress of this approach in oncology, as well as the most recent developments in this area. This is followed by a comprehensive overview of the different imaging modalities that are relevant to lung drug delivery, including nuclear imaging, X-ray imaging, magnetic resonance imaging, optical imaging and mass spectrometry imaging. For each of these modalities, examples from the literature where these techniques have been explored are provided. Finally the different applications of these technologies in oncology are discussed, focusing separately on small molecules and nanomedicines. We hope that this comprehensive review will be informative to the field and will guide the future preclinical and clinical development of this promising drug delivery strategy to maximise its therapeutic potential.

## Graphical abstract

**Figure:**
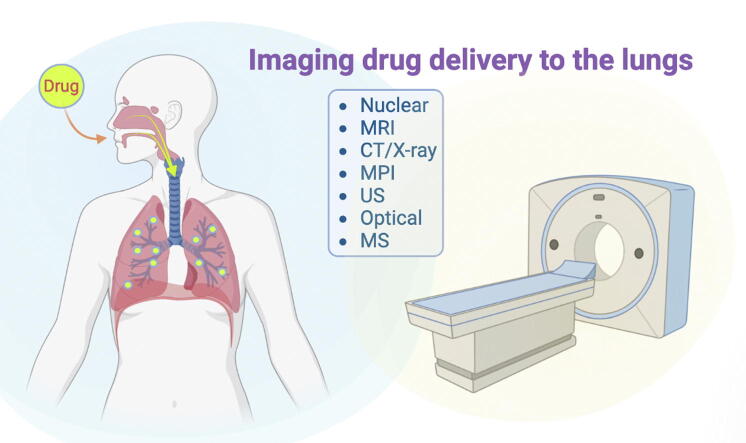

## 1 Introduction

Lung cancer is the most common cause of cancer death, with only 1 in 10 people surviving the disease over 10 years in England.[1] It is also disappointing that survival rates for lung cancer have not improved in the last 40 years, highlighting the clinical need for improved treatment options.

Chemotherapy is the most common option for primary lung cancer treatment, usually via intravenous administration of cis/carboplatin as well as other cytotoxic drugs such as docetaxel and etoposide among others. It is well established that intravenous administration of untargeted drugs leads to a suboptimal accumulation in the disease tissue, as well as severe side effects for the patient due to the systemic exposure or accumulation of the drug in healthy tissues. Taking this into account, direct lung delivery of therapeutic drugs by inhalation appears as arguably the most logical route to treat lung cancer, but attempts to exploit this approach have not progressed effectively in clinical trials.

In this work we focus on lung imaging in order to gain an understanding of this lack of progress by reviewing imaging studies relevant to drug delivery to the lungs. It is becoming clear that integrating imaging studies into the development and clinical evaluation of drug delivery systems allows a comprehensive understanding of their biological behaviour and provides a highly valuable tool to: (i) identify the best systems to progress, and (ii) monitor their efficiency in clinical trials. Different established and new imaging modalities can provide highly valuable information on several important factors that influence the efficiency of drug delivery to the lung, including quantification of deposition and retention, drug release, whole-body biodistribution (BioD) and pharmacokinetics (PK), route and rate of clearance, among others. With this in mind, the aim of this report is to review published studies where preclinical and clinical imaging has been used to evaluate lung drug delivery, and to try to shed some light on the factors that hinder the progress of this approach for the treatment of lung cancer.

## 2 Recent developments in inhaled medicines in oncology

### a Specific challenges for drug delivery in lung cancer

Pulmonary drug delivery is a promising approach for the treatment of localised lung cancer. Inhaled medicines are well established as therapies for managing respiratory diseases such as asthma, chronic obstructive pulmonary disease (COPD), cystic fibrosis (CF), infections, and more recently for the delivery of compounds for systemic exposure (e.g. insulin, loxapine).[2] This non-invasive route of drug delivery has numerous advantages that could be used for efficient and effective lung cancer therapy.[3] Localised chemotherapy has been relatively successful against various types of cancer, including colorectal, ovarian and brain cancers.[4], [5], [6] By administering drugs directly to the lungs, the drug is delivered directly to the target tissue, avoiding first-pass metabolism and plasma binding and systemic distribution.[7] As compounds can be deposited topically on or close to tumours, vascularization of the target is not as relevant as for systemic treatments. This is particularly important in cases of poorly or non-vascularized tumours, which might require even higher doses of drug due to hypoxic environments that favour invasive and more resistant cancer cells.[8], [9] The inhalation route enables smaller doses to be delivered to achieve equivalent lung exposure compared to systemic routes of administration, which reduces the toxicity associated with the exposure of non-target organs to anticancer drugs.[10]

Over the last 30 years, a number of clinical trials have investigated the potential use of aerosolised anticancer therapy.[11], [12], [13], [14], [15], [16], [17], [18], [19], [20] To date, however, none of these clinical trials have progressed beyond phase II and, despite inhaled delivery offering clear pharmacokinetic advantages with significantly reduced side effects compared to systemic delivery, some important issues have been highlighted from those studies.[10] These challenges are discussed in more detail below, but include *(i)* anatomical challenges (*e.g*. lung anatomy and physiology), *(ii)* technical specifications (e.g. inhalation device, formulation strategies, physicochemical properties of drugs, administration time, local contamination), *(iii)* disease stage (*e.g*. tumour size, localisation, partial or complete airway obstruction) and *(iv)* local adverse effects. These aspects play an important role in determining whether aerosolised drug delivery is indeed a feasible and/or effective option for lung cancer therapy.

The structural complexity and inaccessibility of the peripheral lung airspaces, and competing clearance pathways make the inhalation route more complex for drug delivery than most other routes of administration.[21] The fate of respirable particles depends on their deposition site and physicochemical properties. Only the fraction of the emitted dose from an inhalation device that passes the trachea is delivered to the lower airways. This fraction is determined by inhaler device characteristics, patient breathing profile and aerosol particle properties (e.g. aerodynamic diameter < 5 μm, shape). Once deposited, respirable particles may be cleared from the lungs by absorption to the systemic circulation, mucociliary clearance, macrophage phagocytosis or degradation by enzymes.[22], [23]

The airways might be affected by diseases such as narrowed bronchioles in asthma, tumours obstructing the airways, thick and sticky mucosal airways in cystic fibrosis, and inflamed airways in bacterial infections, all of which add more challenges for inhaled drug delivery. These barriers can lower the fraction of emitted dose that can reach the targeted organ, and thereby influence the effectiveness of the therapy.[2] Therefore, suitable aerosol characteristics as well as correct usage of the inhaler are required to obtain optimal drug deposition in the target site in the lungs.

Although there are many different types of inhalation devices in clinical use, to date only nebulisation systems (e.g. using ultrasonic wave, jet, vibrating mesh, breath-enhanced jet) have been used in pilot studies and clinical trials to administer inhaled anticancer compounds.[24], [25] Nebulisers generate droplet aerosols with particle sizes below 5 μm that patients can inhale during normal breathing. The two main challenges with nebulisers are to increase efficiency and portability.[25] Additionally, during the inhalation process a major part of aerosol is lost in the air (e.g. jet nebulisers can have losses of 50 %, with only 10 % of the initial dose deposited in the lungs), which brings further complications in terms of potential exposure of carers and bystanders, particularly in the case of cytotoxic drugs. As the nebulisation of 5 mL of liquid typically takes approximately 10–20 min, the whole process of administration is time-consuming, for example 5-fluorouracil (5-FU) with a dose at 250 mg/5 mL requires delivery twice a day for 15 min, [11] and cisplatin at 1 mg/mL requires 3 sessions per day lasting 20 min each.[19] This leads to airborne environmental contamination that requires to use protective equipment (e.g. respirator face mask, safety glasses, gloves, cap, etc) and time- and resource-consuming procedures. To overcome these issues, dry powder inhalers (DPI) have been suggested as more appropriate devices as they can deliver relatively large doses (up to approximately 400 mg)[26] in a single inhalation effort, thus minimising airborne contamination. Furthermore, powder formulations are more stable compared to liquid formulations and more appropriate for poorly water-soluble compounds. However, the effectiveness of deposited dose in the lungs is dependent on the patient’s inspiratory airflow, another potential source of intra- and inter-patient variability. So far, only a limited number of pilot studies have investigated the use of DPI devices to deliver cytotoxic chemotherapeutic drugs.[10].

A major focus of research in this area is on the development of novel formulations to maximise lung deposition and to minimise clearance of inhaled particles from the lungs. The use of strategies such as sustained-release formulations and the use of liposomes, micelles, microparticles and nanoparticles have been widely investigated to improve both lung tolerance and anti-tumour efficacy. For example, Roa *et al*. showed that by administering inhaled doxorubicin nanoparticles, there was reduced toxicity compared to free doxorubicin in a mouse model.[27] The controlled release properties of some nanoparticle structures may further support the effectiveness of inhaled anticancer therapy by maintaining drug concentrations at tumour site for a longer period.

The impact of tumour stage/progression on the respiratory airflow needs to be more intensively investigated as it has major implications on the effective drug deposition in the lungs and the ability of drugs to reach to site of action. *In silico* modelling has shown that the obstruction of the airways by tumours, depending on the position, might reduce the flow rate and thereby significantly impact the deposition efficacy as most particles were deposited in the upper airways.[28] Finally, a major concern in inhaled drug delivery is potential of the local adverse effects that might develop during drug exposure.

## 3 The role of imaging in inhaled drug delivery

Because the inhaled drug delivery may produce variability in deposited dose, it is important to use methods that can evaluate deposition non-invasively. This is particularly important for drugs that have narrow therapeutic indexes, a delayed therapeutic effect, and in clinical trials so that the therapeutic effect of drugs can be correlated with the actual amount of drug deposited. Non-imaging methods based on ‘downstream’ fluid sampling after the drug has left the lungs (e.g. blood or urine) cannot differentiate between the inhaled and swallowed fraction of the drug, neither do they indicate the regional deposition within the lungs. Notably, studies of aerosol deposition using planar scintigraphy were instrumental in revealing the inhomogeneity and interindividual variability of deposition patterns and greatly assisted the development of improved inhaler devices.[29]

For lung tumours in particular, the impact of the presence of tumours in the airways on the deposition of inhaled drugs is not well understood, and likely to vary significantly between patients. This was illustrated in a study of aerosolised gemcitabine and [^99m^Tc]Tc-DTPA that showed that aerosol distribution was affected by lung ventilation defects due to bronchial tumour or lobectomy,[17] and demonstrated the utility of nuclear imaging in evaluating aerosol deposition. As a useful reminder that inhaled drug delivery is about delivering the right amount of drug to the right place, rather than achieving more uniform or higher total deposition, an interesting preclinical study of inhaled dry powder vaccine formulations showed that an influenza vaccine formulation led to an increase in serum IgG levels irrespective of the deposition pattern – shown by fluorescence imaging – in the airways, whereas for hepatitis B (a blood-borne disease) the IgG levels only increased after deposition deeper in the lungs and, presumably, systemic absorption.[30] This illustrates the importance of tailoring drug delivery to the mechanism of action of the drug, and correlating deposition and tissue/organ retention data obtained by imaging with therapeutic efficacy. In this respect, imaging methods that can track the fate of the drug beyond deposition are especially useful.

Ehrmann *et al.* commented in their recent review article that “Imaging methods are not usually used for pharmacokinetic studies as they do not provide direct quantification of the drug in the lungs and in the systemic compartment.”[31] Whilst this is true of many imaging methods currently used in the clinic, recent developments have improved and facilitated image-based quantification. Advances in lung MRI, further discussed in Section 3.d of this review, now allow absolute quantification of contrast agents in the lungs, although this remains non-trivial. Modern algorithms for single-photon emission computed tomography (SPECT) can more easily quantify the detected signals.[32], [33] PET imaging in particular provides an answer to this challenge, as it allows direct quantification of drugs in multiple organs, simultaneously and with sufficient temporal resolution to enable pharmacokinetic studies to be conducted. The advent of total-body PET will only improve on this, allowing high sensitivity quantitative imaging at the whole-body level.

Imaging drug delivery to the lungs should be considered distinctly from imaging lung structure and function [34], [35] and molecular imaging of the response to drugs or other therapeutic interventions. By structural and functional imaging of the lungs, we refer to techniques such as standard X-ray/CT, MRI, ventilation scans and airflow velocity mapping [36] that are used to inform on the presence of morphological abnormalities, tissue scarring, obstructions, on lung compliance and motion for example. By molecular imaging of the response to treatment, we refer to the imaging of specific biological processes in lung tissue that are indicative of a disease state. This includes for example the imaging of metabolic processes (e.g. glucose consumption with [^18^F]fluorodeoxyglucose ([^18^F]FDG), nucleic acid consumption in proliferating cells with [^18^F]fluorothymidine), of biochemical parameters (e.g. tissue hypoxia with [^64^Cu]Cu-ATSM or [^18^F]fluoromisonidazole) and of cellular markers (tumour antigens, angiogenesis and inflammation markers). In this review we primarily include studies that directly image the drug or delivery system, but we also include a few studies where the responses are a direct indicator of successful drug delivery rather than general improvement in lung condition or disease state. Another frequently used imaging method in preclinical models of cancer is the optical imaging of bioluminescent tumour cells, where the light intensity emitted from the tumours is used as a marker of tumour burden. Although there are numerous examples of tumour and tumour response imaging after inhaled drug administration, these studies are not extensively covered here as the focus of the present review is the imaging of therapeutic molecules and drug delivery systems.

## 4 An overview of imaging methods relevant to lung delivery

In the following subsections we give a brief overview of the principal imaging modalities used for evaluating pulmonary drug delivery, with a description of their underlying principle and selected examples of applications in respiratory diseases. This review primarily focuses on preclinical and human *in vivo* imaging methods. Each modality has significant advantages and drawbacks, and no modality provides the optimal combination of high sensitivity, high spatial and temporal resolution, low cost, wide availability, and patient safety. The choice of an imaging modality should primarily be guided by the type of question the investigator wishes to answer.

### a Challenges in imaging lungs and drug delivery imaging

Imaging drug delivery in the lung presents several specific challenges. As is the case for cardiac imaging, the lung is an organ in constant motion and this motion can introduce artefacts in images, causing blurring and lowering spatial resolution. Moreover, this motion is non-linear, includes rotational and translational movements, and does not affect all areas of the lungs equally. In humans, this can be mitigated to some extent by gating the acquisition, asking the subject to hold their respiration during imaging, using computed tomography for anatomical mapping and using specifically developed MRI sequences. For further details on motion correction we refer the reader to a number of useful reviews.[37], [38], [39] The motion issue is more difficult to avoid in small animals because of their higher respiratory rates, but recent technological improvements now allow gating based on rapid CT imaging for example.[40], [41], [42] Furthermore, animals need to be anaesthetised for imaging, and anaesthesia is a non-physiological state in which respiratory rate is reduced. Thus, imaging drug delivery under anaesthesia may not be fully representative of real-life conditions. Artificial ventilation can be used in animals to standardise imaging conditions but can also cause lung inflammation particularly if used repeatedly or over prolonged periods. For imaging modalities using ionising radiation (X-ray/CT, scintigraphy, SPECT and PET), the radiation dose also has the potential to cause cell damage and affect the immune system.[43], [44] Passive exposure chambers can be used to administer inhaled drugs to animals in more physiological conditions, but these are poorly suitable for imaging purposes. Drug delivery by exposure chambers is inefficient in that only a small fraction of the drug present in the atmosphere will be inhaled, leading to significant wastage of material. A significant amount of drug/tracer may also be taken up orally, or adhere to the skin/fur of the animals. Even though the drug may have no effect in these conditions, it may generate a detectable but artefactual signal in the imaging system. Using a mannequin equipped with a face mask, Haw *et al.* recently demonstrated that a significant amount of aerosol is deposited on the mask itself and the subject’s face,[45] again showing the advantage of imaging over sampling-based methods in quantifying drug deposition on uneven surfaces. Finally, for radiotracers it is also important for operator safety reasons that only the strictly necessary amount of radioactivity be used.

Inhaled drug delivery has its own challenges due to branching and progressively narrowing airways, the presence of mucus as a barrier, and the mucociliary escalator as an efficient clearance mechanism. An optimal imaging agent for drug delivery is one that not only reflects the amount and distribution of drug deposited in the lungs, but is also affected by all these obstacles in the same way as the drug of interest and therefore accurately reflects its fate. The easiest way to achieve this is to covalently attach the imaging moiety (fluorophore, radionuclide, MR/X-ray contrast agent) to the drug. Validation studies are still necessary to verify that the imaging agent (i.e. drug + imaging moiety) has similar affinity for its target to the parent compound, similar lung clearance kinetics, and that it is stable in the intended conditions of use (stability in the formulation for inhalation as well as in lung lining fluid, for example). When a drug is delivered via a macromolecular carrier (e.g. liposome, organic or inorganic nanoparticle, etc), it is important to determine whether the imaging moiety is linked to the carrier or the drug itself. Care must also be taken to ensure that the addition of an imaging agent does not affect the aerodynamic properties or dissolution of the inhaled formulation, which can be difficult for suspensions and powders as the particle size, density, shape, solubility and other characteristics should remain unchanged. Initially, the distribution of the drug will follow that of its carrier, but ultimately with such systems the aim is for the drug to be released from the carrier to reach its intended target. If the imaging moiety is attached to the carrier, subsequent imaging will inform only on the distribution of the carrier. Conversely, if the imaging moiety is attached to the drug, the fate of the carrier after drug release is unknown. The larger issue with this approach is that it often requires chemical modification (such as covalent conjugation to a fluorophore or chelator) and therefore the drug-imaging agent complex is a different chemical species from the drug itself, and there is no guarantee that its biokinetic behaviour will be identical to the parent compound. Imaging approaches that can inform on both the drug and its carrier and demonstrate release are of great interest, but not all imaging modalities are suitable for this.

To measure drug concentration in the lungs, an alternative to imaging is *in vivo* microdialysis. This technique is based on the implantation of a semi-permeable membrane in the organ of interest, the membrane being perfused with a suitable buffer to collect the drug of interest over time. In a recent example, the lung and blood kinetics of an aerosolised antibody were obtained with high accuracy, and imaging was used only to evaluate initial deposition.[46] Amongst the advantages of microdialysis, we highlight that it requires no modification of the drug of interest, and repeated sampling of a subject is possible unlike BAL fluid collection. It is limited however by being a relatively invasive technique that suffers from sampling bias.

### b Nuclear imaging of inhaled drugs and aerosol particles

Nuclear imaging is one of the most common methods to evaluate drug delivery to the lungs, and particularly gamma scintigraphy due to the relatively wide availability of planar gamma cameras and relevant radionuclides (mostly ^99m^Tc). The attenuation of gamma rays by biological tissues is minimal, i.e. their depth of penetration is practically unlimited and radiolabelled drugs located deep inside the lungs can easily be detected. Furthermore, the very high sensitivity of nuclear imaging allows the detection of very low drug concentrations (nanomolar to picomolar range). A drawback of nuclear imaging is that the spatial resolution is limited to 3–7 μm for current clinical gamma cameras. Planar scintigraphy only provides 2-dimensional projections of the distribution of radiolabelled drugs, but this can be overcome by the use of SPECT and PET, which are 3-dimensional techniques. Additionally, PET and modern SPECT reconstruction algorithms allow the accurate quantification of radioactive molecules.[32], [33].

PET and SPECT alone do not provide anatomical information but are usually combined with CT or MRI for this purpose. This allows better tissue delineation to determine drug quantities in individual regions of the lungs. Tissue attenuation correction is particularly important for accurate quantitative measurements. CT is the standard method to perform attenuation correction, but MRI can also be used for this purpose.[47] An inherent disadvantage of nuclear imaging, particularly when combined with CT, is the exposure to ionising radiation, which is all the more relevant in the context of pulmonary delivery as the lungs are one of the more radiosensitive organs.[48], [49] Further improvements in co-registration and tissue segmentation algorithms and patient-specific computational models will also lead to more accurate quantification and localisation of radioactivity in the lungs.[50], [51]

#### Ventilation/perfusion (VQ) scans

The most used nuclear imaging technique in pulmonary medicine is the ventilation/perfusion (VQ) scan, where lung perfusion is imaged using radiolabelled macroaggregated albumin (MAA) and ventilation using a radioactive gas (^133^Xe or ^81m^Kr) or an aerosolised solution ([^99m^Tc]Tc-DTPA) or fine powder (Technegas™, consisting of aerosolised ^99m^Tc-labelled graphite sub-micron particles). This is a functional imaging technique that does not inform on drug delivery but can be used to monitor response to treatment. Occasionally, ventilation studies using inhaled [^18^F]FDG have been performed. The low solubility of nitrogen gas in blood and its diffusion into the alveolar space has also been exploited to perform ventilation and perfusion scans by PET using intravenously administered or inhaled [^13^N]N_2_.[52] More recently, ^68^Ga has been explored as a PET equivalent of ^99m^Tc for VQ scans under the form of ^68^Ga-MAA and ^68^Ga-labelled graphite particles (“Galligas”),[53] and a study by Hofman *et al.* provides a good illustration of the improvement in image quality with PET/CT compared to SPECT/CT.[54]

#### Scintigraphy/SPECT

Scintigraphy and SPECT are based on the detection of gamma rays emitted by decaying nuclei using a gamma camera. These cameras comprise a collimator, one or several detectors, and photomultiplier tubes or photodiodes. The collimator is a block of electron-dense material (lead) traversed by parallel holes, typically in a honeycomb geometry. It filters out gamma rays produced by Compton scattering and those emitted outside of the FOV and helps localise the source of the activity. Only those incident rays that are parallel to the holes are able to reach the detector. In practice, this leads to the attenuation of the vast majority of incident photos and only a small fraction (typically <0.1 %)[55] of the total emissions are effectively captured by the camera, reducing the sensitivity of this technique. The interactions between incident gamma rays and scintillation crystals or semiconductor detectors generate visible photons. These photons are then converted into an electrical signal by the photomultipliers. A planar scintigram can be obtained by a single camera, or two cameras facing each other to simultaneously obtain a frontal and a dorsal image. These images are planar projections of the signal emitted and therefore it is not trivial or even possible to precisely delineate all regions of the lungs. Relatively simple measures of regional deposition, e.g. the penetration index (ratio of radioactivity in peripheral vs central regions), can be made by drawing regions of interest on the images and performing corrections between anterior and posterior images, but there is a lack of standardisation between laboratories. SPECT is performed by rotating the cameras and serially acquiring images and reconstructing a three-dimensional picture from these images. Because gamma-emitting radionuclides emit radiation at defined energy levels and the detector response is dependent on the energy of the incident gamma rays, gamma cameras can image several radionuclides simultaneously by using multiple energy windows. In practice, 2 or 3 radionuclides can be resolved at a time. This is an important advantage of SPECT (and scintigraphy), as it allows the detection of more than one process at a time. For example, Locke *et al.* developed a method making use of ^99m^Tc-sulfur colloid and [^111^In]In-DTPA to simultaneous image mucociliary clearance (affecting both the colloid and DTPA) and liquid absorption (affecting DTPA only) in the lungs, and subtracting one from the other to better determine the rate of liquid absorption in cystic fibrosis and asthmatic patients.[56], [57], [58] Another possibility would be to image inhaled drug combinations labelling one drug with a certain radionuclide and the second one with another radionuclide. It is also possible to radiolabel a drug carrier (e.g. liposomes) and the drug itself, allowing direct imaging of drug release, but this approach adds a degree of complexity and examples of its use are few and far between.[59], [60] To our knowledge, the only reported example of this approach with pulmonary delivery is a study by Thakur *et al.* examining the pulmonary delivery of a tuberculosis subunit vaccine, in which the peptide antigen was radiolabelled with ^67^Ga and the liposomal delivery vehicle with ^111^In, showing more rapid clearance of the antigen from the lungs compared to the liposome.[61]

#### PET

PET is based on the detection of positron annihilation events after the β^+^ decay of a nucleus. Typically, nuclei that have an excess of protons relative to the number of neutrons decay by emitting a β^+^ particle, which is the antimatter counterpart of an electron. Positrons are emitted in random directions and can travel a short distance (up to a few millimetres in tissue), depending on their energy, before colliding with an electron. This annihilation event results in the emission of two 511 keV gamma photons at a near 180° angle from each other. A PET camera is composed of detectors arranged in a circle, enabling the detection of gamma rays from any direction. Collimators are not required, instead the localisation of the original annihilation event is performed by time-based correlation: pairs of photons detected by opposing detectors within a very short time period (<500 ps, depending on the PET camera model) are considered to have originated from the same annihilation event. The absence of collimators means that the number of “useful”, i.e., actually detected photons is far higher than with a gamma camera, and the sensitivity of PET is 2–3 orders of magnitude higher than SPECT. On the other hand, because it is the location of the annihilation event that is computed and not that of the decaying nucleus, there is a fundamental limit to the spatial resolution of PET, which depends on the energy of the positron. Unlike SPECT, standard PET can only be used to image one radionuclide at a time, because all positron annihilation events result in the emission of 511 keV gamma rays. Recently, PET scanners have been developed that are able to discriminate between two PET radionuclides based on the high-energy gamma rays that are also emitted by some of these radionuclides, in effect matching characteristic gamma rays to a pair of 511 keV photons.[62]

#### Spiking aerosols with radiotracers

**Fig. 1:**
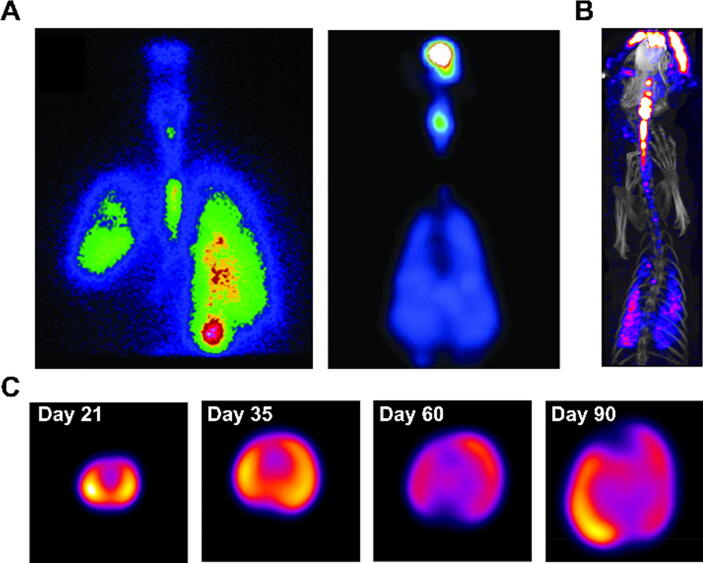
Examples of scintigraphic and SPECT images of inhaled drug formulations. (A) Planar scintigraphic image of [^99m^Tc]Tc-DTPA-gemcitabine aerosol in a patient with right lobar atelectasis (left) and SPECT image of aerosolised lung surfactant radiolabelled with ^99m^Tc-sulfur colloid in a non-human primate (right). Reproduced from Lemarié et al.[17] and Gregory et al.[68] respectively. (B) SPECT/CT image showing the deposition of a [^99m^Tc]Tc-DTPA-labelled dry powder formulation in a ferret. Reproduced with permission from Kuehl et al.[72] (C) SPECT transversal slices of rat lungs aged 21–90 days after inhalation of ^195^Au-labelled gold nanoparticles, showing left-to-right inhomogeneity of deposition across the ages. Modified from Kreyling et al.[73]

**Fig. 2:**
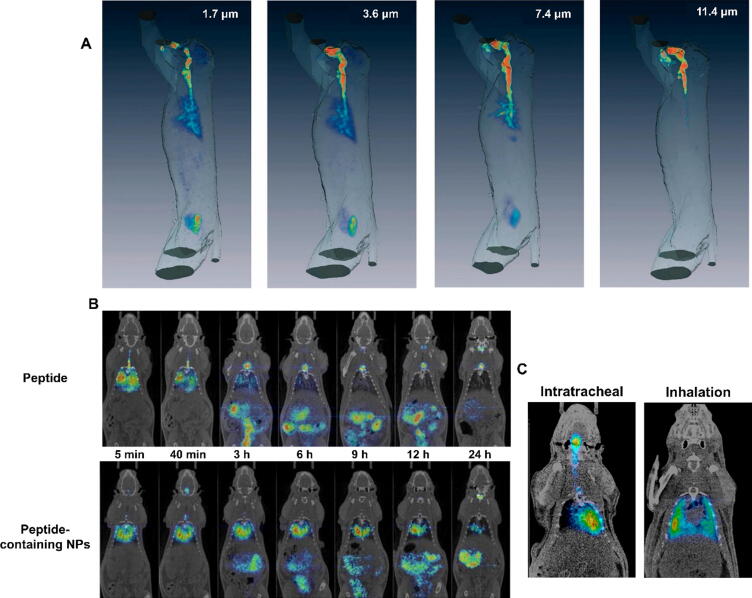
Example PET/CT images of radiolabelled aerosols containing [^18^F]FDG to study particle deposition. (A) PET/CT images showing the difference in deposition patterns of [^18^F]FDG-labelled aerosol droplets of 1.7–11.4 μm aerodynamic diameter in non-human primates. Adapted from Dabisch et al.[66] (B) PET/CT coronal images of a ^124^I-labelled peptide administered intratracheally in solution or bound to dextran nanoparticles. Reproduced from Falciani et al.[99] (C) PET/CT coronal images showing the deposition of [^18^F]FDG in rats immediately after delivery by endotracheal insufflation using a MicroSprayer® Aerosolizer (left) or using an Aeroneb® Micropump Nebulizer device. Adapted from Cossio et al.[65]

For nuclear imaging of inhaled drugs, a frequently used approach is to spike the formulation with a radioactive tracer that will therefore be delivered concomitantly. It is the simplest approach because standard tracers that are available in any radiopharmacy can be added to any solution or suspension for inhalation, without the need to develop radioactive analogues of drugs. As in this case the radionuclide is not covalently bound to the drug, its distribution *in vivo* will not necessarily follow that of the drug and therefore this approach can only be used to image aerosol particle deposition immediately after inhalation, and validation studies are required to determine whether the radioactive signal correlates with the amount of drug deposition (e.g. through tissue or blood sampling). Typical radiotracers for aerosols include [^99m^Tc]Tc-DTPA, ^99m^Tc-pertechnetate and more recently [^18^F]FDG, and this approach is a very convenient and useful way of comparing the performance of aerosol delivery devices and formulations,[63], [64], [65], [66], [67], [68] (Fig. 1, Fig. 2) or simply to provide confirmation and early quantification of deposition in the lungs.[69], [70] For example, Respaud *et al*. nebulised a solution containing an anti-ricin antibody and [^99m^Tc]Tc-DTPA into non-human primates and were able to quantify that 13 ± 7 % of the nebulised dose reached the lungs, whereas 25 ± 7 % of the dose was found in the stomach immediately after inhalation. The limitation of this imaging technique is illustrated by subsequent measurements by ELISA, which showed that the antibody levels in BAL fluid remained near-constant in the 72 hours following inhalation.[71] In contrast, the [^99m^Tc]Tc-DTPA in the solution would have cleared (and decayed) much more rapidly, and later scans would have provided images representative of [^99m^Tc]Tc-DTPA clearance but not of antibody distribution. Another consideration for this approach is that it is easier to radiolabel liquid formulations than powders.

#### Radiolabelled drug delivery to lungs

Nuclear imaging of the biodistribution of drugs and drug delivery vehicles beyond the initial deposition in the lungs requires them to be radiolabelled. This can present an additional challenge as the radiolabelled drug is often chemically different from the parent molecule. Indeed, the most common gamma-emitting radionuclides (e.g. ^99m^Tc, ^111^In, ^123/125^I, ^67^Ga) are metals or halogens that are not part of the elemental composition of typical drugs and need to be added, either directly (for radioiodine, or for ^99m^Tc via reduction) or through a chelator group, thus resulting in a different chemical entity that may behave differently both pharmacokinetically and pharmacodynamically. For PET imaging, ^11^C, ^13^N and ^15^O can sometimes be substituted for carbon, nitrogen and oxygen atoms in drugs, resulting in essentially chemically identical radiotracers. ^18^F can be substituted in drugs that contain fluorine or can frequently be incorporated (as an isostere of hydrogen) into drugs with minimal effects on their properties, but other positron emitters (e.g. ^64^Cu, ^68^Ga, ^89^Zr) can only be incorporated using chelators. This is less of an issue for larger molecules such as antibodies and nanomaterials (micelles, liposomes, nanoparticles, etc.) as the impact of conjugation and radiolabelling on drug physicochemical properties will be lower relative to the overall size of the molecule.

#### PET tracers for lung diseases

[^18^F]FDG PET is by far the most commonly used radiotracer for imaging cancer and inflammation in the lungs, and detects the increase in glucose metabolism of both tumour cells and activated immune cells. It is, however, typically administered intravenously and primarily provides metabolic information.[74] The flip side of this tracer is that increased FDG uptake alone does not differentiate between cancer and inflammation. Many other radiotracers can also be used to image various processes associated with lung (and other) cancers. These can be categorised into metabolic radiotracers based on amino acids (e.g. [^11^C]methionine, L-[3-^18^F]-α-methyltyrosine, [^18^F]fluoroethyltyrosine), nucleic acids (e.g. 3′-deoxy-3′-[^18^F]fluorothymidine, [^11^C]thymidine), lipids or lipid metabolism (e.g. [^11^C]acetate, [^11^C]choline, [^18^F]fluorocholine), radiotracers that detect hypoxia (e.g. [^18^F]fluoromisonidazole, [^18^F]FAZA, [^64^Cu]Cu-ATSM) or apoptosis (radiolabelled annexin V),[75], [76] radiolabelled hormone analogues ([^18^F]fluoroestradiol for oestrogen receptors, [^18^F]fluorodihydrotestosterone for androgen receptors, radiolabelled octreotide analogues for somatostatin receptors), radiotracers based on surface proteins overexpressed in tumour cells (e.g. folate receptors, radiolabelled RGD peptides for integrin α_V_β3, [^18^F]fluoroDOPA for brain tumours, ^68^Ga-labelled fibroblast activation protein (FAP) inhibitors,[77], [78] and the tumour micro-environment (e.g. ^64^Cu-labelled nanoparticles to image tumour-associated macrophages.[79] Numerous PET and SPECT radiotracers have also been developed with the aim of imaging non-malignant lung diseases, for example [^64^Cu]Cu-PEG-FLFLFK to image neutrophils in lung infections,[80], [81], [82] ^68^Ga-labelled nanoparticles for neutrophils in lung inflammation,[83] ^64^Cu-labelled integrin α_v_β_6_- targeting peptides for idiopathic pulmonary fibrosis and lung cancer,[84] ^99m^Tc-labelled interleukin 8 for lung infections.[85] Recently, ^68^Ga- and ^18^F-labelled FAP inhibitors have shown promising results for the imaging of activated fibroblasts in interstitial lung diseases.[86], [87] These tracers are usually administered intravenously and require the target area to be sufficiently vascularised to accumulate. For further information we refer the reader to review by Szyszko *et al*.[88]

#### Imaging of inhaled nanoparticles

Several studies have used radiolabelling and nuclear imaging to study the deposition and kinetics of microparticles and nanoparticles in the lungs, investigating the influence of parameters such as particle size, material, administration method, but also the developmental stage[89] or disease status of the recipient lungs. For gold nanoparticles <100 nm administered intratracheally, the translocation across the epithelial barrier was inversely proportional to the particle size.[89] Inhaled ^192^Ir nanoparticles (15/80 nm) were mostly cleared through the larynx, i.e. by mucociliary clearance, followed by gastrointestinal (GI) elimination, with <1 % of the particles translocating from the lungs into the circulation and secondary organs.[90], [91] Overall, there was little difference between gold and iridium nanoparticles, and between nanoparticles administered by inhalation or intratracheal instillation.[73] Studies of ^48^V-labelled titanium dioxide nanoparticles and ^105^Ag silver nanoparticles were also performed, but without imaging.[92], [93] Although the sensitivity of radioactivity measurements was exploited to establish the clearance pathways of nanoparticles (i.e. macrophage-mediated removal from the alveolar space across the epithelium, first into the interstitial space and subsequently back to the epithelium and into the larynx and GI tract),[94] these data were obtained from *ex vivo* gamma-counting and not imaging. Indeed, the spatial resolution of current gamma cameras limits the level of detail to the distribution of radioactive material between different “large” subsections of lung lobes (e.g. upper vs lower, peripheral vs central) but cannot distinguish between the alveolar, epithelial and subepithelial space, or the type of cell involved in particle clearance. The similarity of nanoparticle kinetics after inhalation and intranasal instillation observed by Kreyling *et al*. suggests relatively homogenous delivery was achieved.[73] The homogeneity of deposition in small animals is known to depend on the techniques and instrument used, as well as operator skill.[65], [95] Using ^76^Br- and ^125^I-labelled polyacrylamide particles in lung inflammation models, it was shown that microparticles (approx. 2 μm hydrated diameter) delivered intratracheally were removed by mucociliary clearance, whereas nanoparticles (31 nm diameter) of the same material had prolonged retention. Coating the particles with a positively charged cell-penetrating peptide further reduced lung uptake of the microparticles but increase retention of the nanoparticles. Additional *ex vivo* investigations with fluorescently labelled particles showed that the particles were retained primarily by alveolar macrophages but also by alveolar epithelial cells.[96] Similarly, salbutamol-containing 60 nm nanoparticles radiolabelled with ^99m^Tc showed deeper penetration into the lungs than the micron-sized particles of the same drug,[97] although mucociliary clearance was also visible 3 h after inhalation. Nanoparticles are often studied as controlled-release drug delivery systems, on the basis that prolonged drug release or retention at the site of action should improve therapeutic activity. A notable study by Weers *et al.* using a nebulised suspension of ^99m^Tc-labelled amikacin-loaded liposomes (now clinically available as Arikayce®) showed the prolonged retention of the liposomes in the lungs of healthy volunteers by planar scintigraphy. The partial clearance of the liposomes through the mucociliary escalator was also observed in the form of radioactive signal originating from the stomach. In this case, the radionuclide was bound to the liposomal membrane and therefore it is the delivery vehicle that was imaged.[98] A study of nebulised antimicrobial nanoparticles in rats took the opposite approach, first radiolabelling the antimicrobial peptide with ^124^I and then grafting them onto 18 nm dextran nanoparticles by electrostatic interaction.[99] The PET/CT images clearly show the increased retention of radioactivity in the lungs, with a biological half-life of 12 h compared to 1 h for the free peptide (Fig. 2B), although the antimicrobial activity was not significantly improved in that model. Conversely, the high uptake and long retention of inhaled ^111^In-labelled polyhexamethylene guanidine (PHMG) particles (0.27 μm) in the lungs have been suggested as an explanation for the high toxicity of PHMG, a biocidal agent found in certain household disinfectants that has caused fatal lung injuries in South Korea.[100] Nanoparticles for drug delivery have seen more use in the context of cancer, and studies investigating the inhaled delivery of nanoparticles against cancer are covered in Section 4.b of this review.

#### Indirect labelling and autoradiography

Drug delivery can be imaged indirectly by using a radiolabelled ligand of a particular receptor and displacing it with the unlabelled drug of interest, effectively performing *in vivo* radioligand-binding studies. For example, the inhaled delivery of ipratropium, a muscarinic receptor antagonist used in asthma treatment, was evaluated in primates using a selective ^11^C-labelled muscarinic radioligand injected intravenously. This study showed that at equivalent plasma concentration of ipratropium, the inhalation route achieved higher target receptor occupancy than the intravenous route.[101] Similarly, target engagement of an inhaled integrin α_v_β_6_ inhibitor was confirmed by PET/CT in patients with idiopathic pulmonary fibrosis (IPF) and by SPECT/CT in a mouse model of IPF using ^18^F- and ^111^In-labelled α_v_β_6_-selective peptides, respectively, showing a reduction in radiotracer uptake 30–120 min after administration of the inhaled α_v_β_6_ inhibitor.[102], [103] This is an interesting approach as it does not require labelling (and therefore modifying) the drug of interest and allows the use of intravenously administered radiotracers, which is easier than preparing radioactive material for inhalation.

An alternative to SPECT and PET for nuclear imaging is the use of whole-body autoradiography.[104] An entire animal can be frozen and cryosectioned to obtain relatively details images of the localisation of administered radioactivity. This was used to demonstrate the progressive release of ^14^C-labelled doxorubicin from an effervescent formulation administered as a dry powder in a mouse xenograft model of lung cancer. The radiolabelled doxorubicin can be seen deposited in a small area of the lungs 1 h after inhalation, and progressively distributing throughout the lungs over 24 h with limited redistribution into extra-pulmonary organs (primarily the intestines), unlike intravenously administered doxorubicin which was found in most tissues.[105]

### c Pulmonary drug delivery imaging by X-ray computed tomography (CT)

X-ray imaging is based on the absorption of X-rays (typical energy range: 20–150 keV) by materials and has been used for medical applications for over 125 years. It is generally used to provide structural information about the body, using the different in electron density between different types of tissue. Bones and teeth are the most electron-dense tissues due to their high calcium content, whereas air-filled lungs have very low X-ray absorption. This makes the lung a more challenging organ to image by conventional X-ray. X-ray imaging can be done in planar mode (comparable to photography) with the subject maintained between an X-ray source and a detector. Since the development of computed tomography (CT) in the 1970′s,[106] X-ray can also be done in three dimensions. In medical CT, the source and the detector rotate around the subject, and a 3D image is reconstructed from multiple 2D images. As changes in lung water content and tissue composition affect X-ray attenuation, CT can be used to detect oedema, fibrosis and many other lung diseases.[107], [108], [109] High-resolution CT imaging can also be used to evaluate airway geometry and develop *in silico* models for computational fluid dynamic analysis of drug deposition in the lungs.[110]

Drugs or contrast agents that are sufficiently electron-dense can be visualised by X-ray imaging and this long been exploited with barium or iodine. For example, iopamidol, iohexol and diatrizoate are iodinated contrast agents used for angiography and urography, and barium sulfate is often used for gastrointestinal imaging. For a more detailed overview of X-ray contrast agents, we refer the reader to a review by Lusic and Grinstaff*.*[111] More recently, X-ray imaging of nanomaterials has also been performed. Indeed, (nano)particles can be made from electron-dense materials such as gold and other metals,[112] or conjugated to electron-dense elements such as iodine. Gold nanoparticles are particularly useful for X-ray imaging because of their high absorption coefficients.

**Fig. 3:**
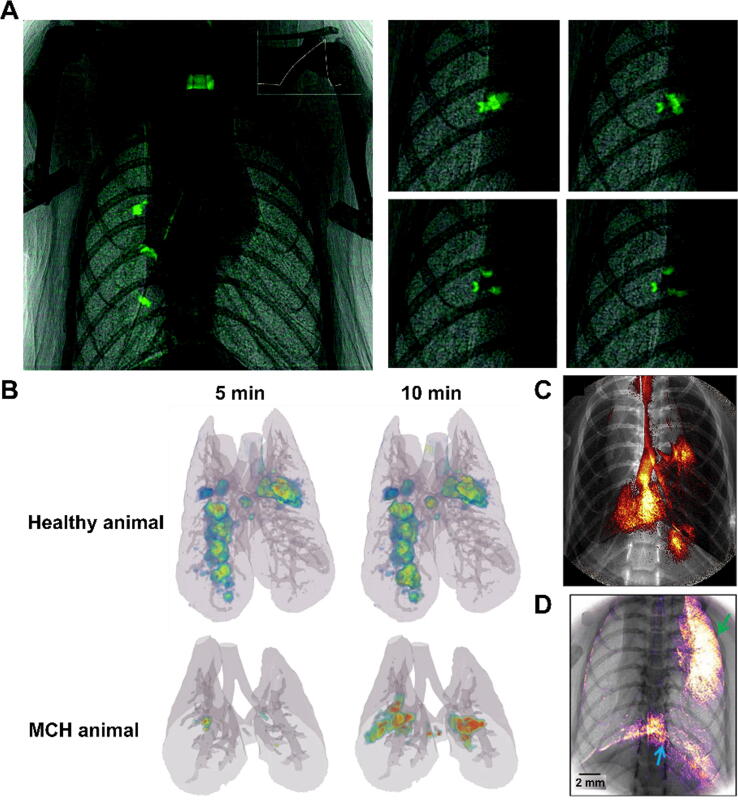
Phase-contrast X-ray imaging after inhaled delivery. (A) PCXI images showing the overall deposition of a 30 μL bolus of iodine-containing solution delivered intranasally in a mouse, 5 breaths after delivery (left) and the splitting of a 30 μL bolus at an airway trifurcation over the course of 1200 ms, approximately 10 breaths after delivery. Adapted from Donnelley et al.[116] (B) Three-dimensional reconstruction of K-edge subtraction PCXI images of the distribution of an iodine-containing aerosol in healthy and methacholine (MCH)-challenged rabbits, showing heterogenous deposition in both subjects but reduced overall deposition in the MCH-challenged subject. Modified from Porra et al.[118] (C,D) PCXI images of iodinated nanoparticles instilled into the lungs. Modified from Gradl et al.[119] and Yang et al.[120] respectively.

Benefitting from highly coherent X-rays produced by a synchrotron, phase-contrast X-ray imaging (PCXI) provides high spatial and temporal resolution, as well as exposure times as short as 100 ms.[113] This reduces motion artefacts that may blur the images and enables real-time imaging of pulmonary delivered drugs. It has been applied to image the deposition of pollutants such as asbestos and quarry dust.[114], [115] For drug delivery, Donnelley *et al.* were able to image the intranasal delivery of small droplets of a solution of iodinated contrast agent to mimic a gene delivery formulation, providing highly detailed images of liquid movement in the nasal airways and upper trachea in mice (Fig. 3A).[116] A small volume of 4 μL of liquid iodine was delivered as a surrogate for lysophosphatidylcholine (investigated as a transfection-facilitating agent) and remained in the treated nasal cavity immediately after delivery. A larger volume of liquid iodine representing the gene vectors was then administered, which overwhelmed the nasal airway and reached the nasopharynx as indicated by dynamic PCXI. The study also investigated the impact of instillation rate on lung delivery, showing that a fast bolus instillation of the contrast agent resulted in more deposition in small airways than slower delivery, although inter-individual variability was high.[116] Accuracy improvements can be obtained by K-edge subtraction (KES) CT, a technique that uses two energies to irradiate the sample, enabling the separation of the contrast agent from endogenous calcium on the resulting images.[117] KES CT has been used to image and quantify the regional deposition of iodine-containing aerosols in rabbits, revealing anatomy information and drug deposition in the same image (Fig. 3B).[118] In both healthy and methacholine-challenged rabbits, inhomogeneous aerosol deposition, indicated as “hot spots” in 2D KES imaging, was observed and prolonged inhalation time increased deposition in lungs. However, narrowed airways in the asthmatic group showed significantly lower contrast deposition compared to healthy rabbits.

Despite the technical advantages of PCXI over conventional CT, the small number of synchrotron facilities in the world, their usage cost and the high radiation exposure of the subject are major obstacles to the use of PCXI. Clinical use of PCXI has not yet been achieved, but the development of more compact synchrotron light sources may bring this prospect closer to reality. Using an inverse Compton scattering-based X-ray source, which is much smaller than a synchrotron and can be installed in a more standard laboratory environment, Gradl *et al.* imaged lung motion and intranasal delivery of iodinated liquids and nanoparticles of various composition in mice,[121] showing for example the formation of bubbles in the airways after inhalation of a nebulised liquid.[119], [120] Because the exposure time (<200 ms) was shorter than a complete breath cycle, the system could be set to capture images at the same point during each cycle, thus minimising the effects of lung motion and providing good delineation of the lung boundaries, enabling dynamic tracking of liquid delivery in free-breathing animals (Fig. 3C,D). In these studies, optical imaging, either through *ex vivo* whole-lung fluorescence or light sheet fluorescence microscopy, provided an additional degree of spatial information whereas PCXI provided the high temporal resolution.

### d Magnetic Resonance Imaging (MRI) of inhaled drugs

MRI is an imaging modality that relies on the magnetic properties of certain nuclei, most frequently hydrogen (^1^H) nuclei in medical applications due to the abundance of water in tissues. By applying a strong, homogenous external magnetic field B_0_ (typically 1.5 T and up to 7.0 T in clinical MRI scanners), the magnetic moments of the nuclei align parallel or anti-parallel to the direction of the field. A radiofrequency pulse, or oscillating magnetic field, B_1_ is then applied at an angle to B_0_ and perturbs the alignment of the magnetic moments, providing magnetic excitation. The magnetic moments then return to equilibrium through a rotating motion, inducing a voltage in a radiofrequency receiver coil placed near the subject or sample. Spatial information can be recovered by applying additional external magnetic fields with spatial gradients. By selecting slices and using phase and frequency encoding, the resulting signals can then be converted into an image. The T_1_ and T_2_ relaxation times (corresponding to the relaxation of the longitudinal and transverse components of the magnetisation vector relative to the external field, respectively) are affected by neighbouring nuclei, in other words the relaxation of each individual nucleus depends on its environment. Thus, contrast can be generated between tissues that contain different amounts of water or fat. In the context of lung imaging, ^1^H-MRI signals correlate with changes in water content in the lungs and can be used as a functional imaging method to visualise processes such as mucus secretion, oedema, drug-induced injury, emphysema and vascular remodelling.[122] These changes have been shown to reverse after treatment with anti-inflammatory drugs, meaning that MRI is a useful technique for imaging response to treatment. Compared to other tissues, the lung parenchyma contains less water, reducing MRI signal intensity. Additionally, the complex air-tissue interfaces and cardiac and respiratory motion can cause imaging artifacts. Because of the absence of ionising radiation, MRI is often used as an alternative to nuclear imaging in “at-risk” patient populations such as infants and pregnant women.

MRI can be enriched by the use of contrast agents. Contrast agents based on gadolinium are paramagnetic and reduce the T_1_ and T_2_ relaxation times of ^1^H nuclei of water in the lung parenchyma and can be used to measure perfusion and ventilation by MRI. Dynamic contrast-enhanced (DCE)-MRI can detect changes in vascular permeability. To image drug delivery, the molecules of interest need to generate sufficient MR contrast to be detectable. This is typically achieved by labelling molecules with paramagnetic ions that increase MR contrast, such as Gd(III) or Mn(II). As these metals are not typically present in drugs, they can be introduced *via* chelators just like the radiometals mentioned in Section 3.b. Another option is to use superparamagnetic iron oxide nanoparticles (SPIOs), which generate strong MR contrast and can be decorated with pharmacologically active molecules or used as drug delivery vehicles. An alternative to ^1^H-MRI for lung imaging is the use of ^19^F-based tracers, which have no intrinsic background and thus provide high-contrast images. The use of ^19^F-MRI for lung imaging is primarily done with fluorinated gases for lung function imaging,[123] but we discuss a few examples of ^19^F-labelled drug delivery imaging in Section 4.b. For more details on the chemistry of MRI contrast agents, we refer the reader to a comprehensive review by Wahsner *et al.*[124] Compared to nuclear imaging, the sensitivity of MRI is several orders of magnitude lower (μM-mM) and can be a limitation to its use in patients. Achieving such concentrations in the lungs requires the administration of hundreds of milligrams of contrast agents, whereas typical doses of inhaled drugs are of less than a milligram. This lower sensitivity means that only relatively large changes in drug amounts can be detected, and at the concentrations required to obtain a good signal, MR contrast agents may also have pharmacological effects. Aerosolised gadolinium-diethylenetriamine pentaacetic acid (Gd-DTPA) has been used in several preclinical models to evaluate lung ventilation by MRI,[125], [126], [127], [128] but has only been reported once in humans, showing homogenous deposition in the lungs of healthy volunteers.[129] A few preclinical studies exist that more specifically examined drug delivery by MRI using Gd-DTPA [130] and SPIOs.[131], [132]

**Fig. 4:**
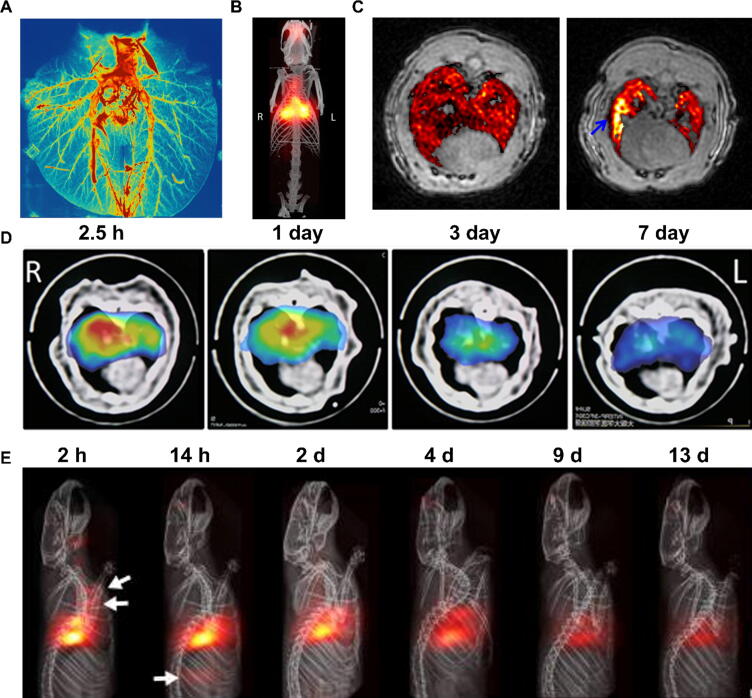
Examples of magnetic resonance imaging of pulmonary drug delivery**.** (A) T_1_-weighted UTE-MRI image (maximal intensity projection) of an *ex vivo* porcine lung after nebulisation of gadolinium-containing polysiloxane nanoparticles, showing up to 8 generations of bronchi. Reproduced from Montigaud et al.[139] (B) Whole-body MPI/CT image of nebulized iron oxide nanoparticles in mice. Adapted with permission from Tay et al.[145] (C) UTE MRI images and concentration map (red signal) pre- and post-administration of aerosolized Gd-DOTA showing hotspots (blue arrow) in an apical region. Adapted from Wang et al.[137] (D) MPI/CT transverse sections of mice after administration of magnetic nanoparticles (Resovist™) showing the progressive clearance of nanoparticles. Adapted with permission from Nishimoto et al.[146] (E) MPI/CT images of mice administered nebulized Perimag™, showing accumulation of the iron nanoparticles in the lungs as well as mucociliary clearance (white arrows indicate the trachea and gastrointestinal tract). Adapted with permission from Tay et al.[145]. (For interpretation of the references to colour in this figure legend, the reader is referred to the web version of this article.)

An issue with the use of MRI for drug delivery evaluation is that concentrations of MRI contrast agents are not easily quantifiable from MR images, although this can be achieved with appropriate methods. For example, Oakes *et al.* quantified inhaled iron oxide nanoparticles in rat lungs *ex vivo* using R_2_* decay rate measurements and showed that particle deposition was higher in the lung periphery than in central airways,[133] and higher and more heterogeneously distributed in elastase-induced pulmonary emphysema compared to healthy lungs.[134] Nanoparticle concentration and pharmacokinetic measurements have also been obtained using ultra-short echo time (UTE) MRI,[135] improving delineation of lung fibrosis compared to contrast-free imaging.[136] UTE MRI has also been used with Gd-DTPA, showing lower deposition of the aerosol in the lungs of ovalbumin-sensitised rats (Fig. 4C).[137], [138] A major advantage of MRI over nuclear imaging modalities is that is has a much higher spatial resolution, which allows for a much more precise determination of the localisation of inhaled drugs. This is particularly relevant in the lungs as the distribution of inhaled drugs between different types of airways and the homogeneity of deposition can affect their PK and pharmacodynamics (PD), which is highly important for directing anticancer drugs to localised tumours. A good illustration of this difference in spatial resolution was provided in recent a study using aerosolized ^111^In-labelled gadolinium-coated nanoparticles in *ex vivo* porcine lungs, where the MRI shows particles up to the eight generation of bronchi (Fig. 4A) and starkly contrasts with the typically low resolution of gamma camera images.[139] It is important to note that being able to image 8 generations is still limited, as models (Weibel’s) suggest up to 23 generations.

As previously mentioned, a major drawback of MRI is the low sensitivity of this imaging modality, particularly compared to nuclear and optical imaging, requiring drug concentrations in the millimolar range. One way to overcome this limitation is the use of hyperpolarised MRI.[140], [141] In this technique, nuclear spin polarisation is increased (e.g. by dynamic nuclear polarisation or optical pumping) by up to 5 orders of magnitude, resulting in MRI contrast agents with very high signal-to-noise ratios, albeit transiently. For examples, the hyperpolarised gases ^3^He and ^129^Xe can be used for lung function imaging by MR, improving temporal and spatial resolution over scintigraphy with ^133^Xe and ^99m^Tc-DTPA.[142], [143] These can be used for ventilation imaging, oxygen partial pressure measurement or gas exchange and could be used to monitor the efficacy of inhaled drugs. There is also considerable interest in using hyperpolarised MRI of biologically nuclei such as ^13^C, ^15^N and ^31^P, which can be incorporated into biological molecules and drugs. In particular, metabolic imaging of the lung can be performed with hyperpolarised ^13^C-labelled amino acids and metabolites such as [1-^13^C]pyruvate, and we refer the interested reader to an excellent review of this field by Siddiqui *et al.*[144] However, the short half-life of the hyperpolarised state of these nuclei (around 20–30 s for ^13^C and a few minutes for ^15^N *in vivo*) is a limitation to this technique. To the best of our knowledge, no studies of inhaled drug delivery using hyperpolarised MRI have been published to date.

A more recent imaging modality related to MRI is magnetic particle imaging (MPI), which is based on the selective excitation of magnetic moments of superparamagnetic metallic nanoparticles, typically SPIOs. Briefly, a time-varying homogenous magnetic field (the selection field) is applied that saturates the particles in the fields, except those in a central field-free region (FFR). A second field, called the drive field is then applied, to which only the particles in the FFR can respond and generate a signal in the receiving coil. An image can then be generated by moving (raster scanning) the FFR in 3D across the sample.[147] In general, the main advantages of MPI over standard MRI are the absence of background signal, a higher sensitivity (nM range) and the ease of quantification. For lung imaging specifically, MPI overcomes the issue of low proton density since the nanoparticles are imaged directly rather than their effects on surrounding protons. Compared to radiolabelled nanoparticles, MPI does not involve ionising radiation and the signal does not decay, allowing longer tracking. However, perhaps because of the novelty of this technique, relatively few research centres are equipped with MPI scanners and thus applications of MPI to pulmonary delivery have been limited to date. Nishimoto *et al*. performed a proof-of-concept study where SPIOs were delivered to mice by nebulisation, enabling the visualisation of these NPs in the lungs and the calculation of their mucociliary clearance rate (Fig. 4D).[146] Similarly, Tay *et al*. added SPIOs to a solution of doxorubicin and nebulised the mixture into healthy rats, showing that the MPI signal from the SPIOs correlated well with the fluorescence from the doxorubicin and therefore that MPI could be used to quantify drug deposition *in vivo* (Fig. 4B,E).[145] With MPI, adding SPIOs to a nebulised formulation is the equivalent of spiking a formulation with [^99m^Tc]Tc-DTPA, in that it is a simple way of evaluating deposition but does not provide drug delivery information beyond deposition. This may become a useful alternative to nuclear imaging of inhaled drug deposition when MPI eventually reaches the clinic.

### e In vivo optical imaging of inhaled drug delivery

**Fig. 5:**
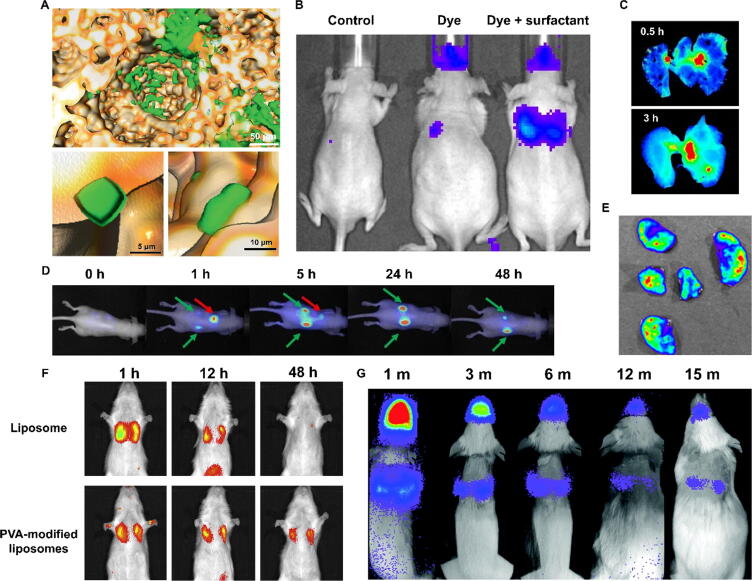
Examples of fluorescence imaging of inhaled drugs. (A) Three-dimensional rendering of mouse lung alveoli (yellow) by micro-optical sectioning tomography, showing the deposition of inhaled cubic metallic-organic framework microparticles (green) as single particles and aggregates. Adapted from Sun et al.[164] (B) *In vivo* fluorescence images showing the distribution of a small-molecule near-infrared dye (Xenolight DiR) alone or in the presence of surfactant, after intratracheal injection. Reproduced from Huang et al.[148] (C) *Ex vivo* images of human serum albumin-coated PLGA microparticles after insufflation in mice, showing progressive deposition throughout the lungs. Adapted from Kim et al.[165] (D) Dynamic tracking of Cy5.5-labelled polysiloxane nanoparticles in mice after intratracheal administration showing uptake in the lungs (red arrow) followed by renal elimination (green arrows). Adapted from Bianchi et al.[135] (E) *Ex vivo* bioluminescence imaging of mouse lung lobes after nebulisation of luciferase-encoding mRNA polyplexes, showing the effective transduction in the lungs following inhaled delivery. Adapted from Patel et al.[166] (F) Deposition of inhaled indocyanine green-labelled submicron liposomes modified with and without a polyvinyl alcohol (PVA) hydrophobic anchor, showing longer retention in the lungs after PVA modification. Adapted from Murata et al.[154] (G) BLI imaging of mice showing the expression of firefly luciferase over 15 months after intratracheal administration of recombinant adeno-associated viral vectors. Reproduced from Vidovic et al.[167] (For interpretation of the references to colour in this figure legend, the reader is referred to the web version of this article.)

Optical imaging is based on the detection of photons in the visible wavelength range, i.e. approximately 400 to 800 nm. The emitted light originates from the de-excitation of molecules returning from an excited state to a lower energy state. The initial excitation can be provided by an incident beam of light of higher energy (in the case of fluorescence), a chemical reaction (for chemiluminescence) or an enzymatic reaction (bioluminescence). In this section we focus primarily on *in vivo* optical imaging plus selected examples of *ex vivo* imaging of inhaled drug delivery. The main issue with *in vivo* optical imaging of the lungs is the limited depth of penetration of visible light (a few mm to a few cm, depending on wavelength), particularly below 600 nm due to the absorption of light by haemoglobin and light scattering by various tissues. In practice, *in vivo* optical imaging of the lungs is limited to small-animal applications with near-infrared fluorophores, and to invasive procedures such as fluorescence bronchoscopy in patients. Illustrating one of the issues with imaging the lungs *in vivo*, a study investigating the use of lung surfactant as a means of improving delivery of budesonide to the lungs also showed a distinct loss of signal intensity and spatial resolution compared to *ex vivo* imaging due to light absorption and scattering by surrounding tissue (Fig. 5B).[148] Similarly, another study in which liquid iodine and a fluorescent dye were co-instilled showed discrepancies between the *ex vivo* fluorescence and CT images, potentially due to signal attenuation of the dye present in the deeper areas of the lung.[119] On the other hand, an advantage of 2D *in vivo* imaging especially compared to nuclear imaging and MRI is a relatively short acquisition time (<1 min) that allows qualitative or semi-quantitative assessment of drug delivery at early time points, as illustrated by the progressive deposition of indocyanine green in rat lungs over 30 min after administration as a dry powder formulation.[149]

The last decade has seen an expansion in the development and availability of fluorescent tracers emitting in the near-infrared (NIR) region (650 nm and above), driven by the rationale that these wavelengths are less absorbed by tissue and therefore provide an increase in penetration depth, facilitating whole-body imaging of mice and rats. NIR tracers have been used extensively to study cancer,[150] vascular diseases,[151] CNS diseases,[152] for intraoperative fluorescence imaging,[153] and in a smaller number of studies of pulmonary drug delivery. For example, NIR imaging has shown that liposomes functionalised with polyvinyl alcohol anchors had significantly longer retention in the lungs after intratracheal administration in rats, with fluorescence in the lungs still observable after 48 h, compared to unmodified liposomes which showed no fluorescence 48 h after administration (Fig. 5F),[154] and that polystyrene nanoparticles had higher uptake in the lungs in a mouse model of ovalbumin-induced asthma compared to healthy mice due to increased phagocyte activity.[155] Inhaled calcium carbonate microparticles 0.65 μm to 3.15 μm in diameter labelled with fluorescent albumin were shown to increase the retention of the Cy7 dye in the lungs compared to the free dye, particularly for the 0.65 μm particles, although in this study the imaging was conducted *ex vivo*.[156] Illustrating the interplay of particle and administration device characteristics in drug deposition, a study using fluorescent spray-dried vaccine formulations showed that powder velocity was a major factor affecting deposition as particles delivered at high speed through an insufflator remained in the trachea or were exhaled, whereas those delivered at a 10-fold lower velocity using an aerosol generator were deposited more uniformly across the peripheral lungs.[30] Overall, these studies show that 2D fluorescence imaging, particularly with NIR tracers, is a viable alternative to nuclear imaging for pulmonary drug delivery in mice and even rats, especially if accurate quantification is not the priority. It is nonetheless a highly useful modality for preclinical studies due to its high spatial resolution especially *ex vivo*, high sensitivity and multiplexing capacity (i.e. imaging multiple tracers simultaneously).

The increasing availability of fluorescence molecular tomography (FMT, also known as fluorescence emission computed tomography, FLECT) that allows quantitative 3D imaging, is an exciting development that should facilitate preclinical imaging of drug delivery in coming years. The way an FMT instrument works is a laser beam is shone through the animal to excite one or more fluorophores, and the transmitted light is collected at multiple angles. The laser is then moved along a grid (raster scan) and the imaging repeated until the area of interest has been scanned. A 3D image is then reconstructed from the different projections acquired. For example, FMT was used with fluorescently labelled ovalbumin to evaluate a new aerosol generator for mice, with the images showing aerosol deposition in the trachea and lungs.[157] Combining FMT with X-ray CT for anatomical registration greatly improves the quality of images.[158], [159], [160] FMT has been used to image pulmonary inflammation and the response to inhaled anti-inflammatory drugs using cathepsin-activatable near-infrared tracers,[161], [162] although these tracers are typically administered intravenously. Recently, Megia-Fernandez *et al.* imaged a matrix metalloproteinase (MMP)-activated fluorescent tracer in the lungs of human patients with pulmonary fibrosis, using a specially developed bronchoscopy catheter to both deliver the tracer and image the lungs, and were thus able to visualise the effect of an endobronchially delivered MMP inhibitor *in situ*.[163]

Three-dimensional imaging of the lungs has also been performed by cryosectioning animal lungs and scanning individual slices. Bauer *et al.* exposed mice from the most commonly used strains in pulmonary research to aerosolised fluorescent microparticles and used the slices to reconstruct highly detailed 3D computational models of the lungs for *in silico* studies of particle deposition,[168] and indeed that dataset has since been used by another group to show that for microparticles in the 0.5–2 μm size range, regional particle deposition heterogeneity, evidenced by deposition hotspots in the cranial lobes of the lungs, increases with particle size.[169] The cryosectioning approach was also used for the quantitative measurement of intratracheally administered fluorescent drugs, with a detection limit lower than 10 ng for a fluorescently-labelled therapeutic protein.[170] Alternatively, tissue clearing of lungs, i.e. the treatment of excised lungs to make them transparent, has recently enabled the quantification and 3D mapping of inhaled nanoparticles within individual alveoli at tissue depths of up to 100 μm by laser scanning confocal microscopy[171], [172] and 2 mm deep by light sheet fluorescence microscopy.[120], [173] By integrating tissue clearing with micro-optical sectioning tomography (MOST), the single-particle tracking of inhaled fluorescent particles within alveoli has also been achieved, showing the deposition of single particles as well as aggregates (Fig. 5A).[164]

To evaluate the delivery of nucleic acids, reporter gene imaging is a powerful tool in preclinical research. Bioluminescent reporter genes are often chosen in this context because of the excellent contrast offered by bioluminescence imaging (BLI), as there no intrinsic background signal. BLI is based on the emission of light by a biochemical reaction, such as the oxidation of luciferin by a luciferase. For use as a reporting system, a sequence coding for a luciferase is typically fused to the sequence coding for the protein of interest in a transfection vector. The luciferase will then be expressed along with the protein and thus the delivery and successful translation of the encoded protein can be assessed. For example, a recombinant adeno-associated viral vector (rAAV) modified with a luciferase gene was tracked over 15 months after intranasal instillation in mice (Fig. 5G), nearly reaching the maximum life span of model animals.[167] The BLI signal showed a strong correlation with rAAV genome copies; rAAV transfection areas decreased gradually in lung and nose, but both remained significantly higher than background signal.

The clinical success of mRNA-based vaccines against SARS-CoV2 has brought mRNA-based therapy into the spotlight. The fragility of mRNA molecules creates an additional challenge for their delivery. A few recent studies have made use of molecular imaging tools to evaluate mRNA delivery to the respiratory tract. For example, Qiu *et al*. developed a PEGylated cationic peptide that forms a nanosized complex with mRNA through electrostatic interactions and allows it to be formulated as a dry powder.[174] Dry powder formulations are attractive for nucleic acid delivery as they provide additional stability to degradation and contamination compared to an aqueous environment. Bioluminescent imaging revealed that the PEG-peptide-mRNA complex was delivered deeper into the lungs than with other transfection vectors. Other studies have investigated cationic polyplexes[166] and lipid nanoparticles as delivery vehicles for mRNA (Fig. 5E), showing for example that delivery through a vibrating mesh nebuliser did not adversely affect *in vivo* transfection efficacy[175] and that changing the structural lipid of nanoparticles could have a tissue-dependent impact on transfection efficacy. Notably, all these studies used firefly luciferase in combination with d-luciferin and imaging of the lungs was only performed *ex vivo.* The emission spectrum of d-luciferin peaks at 562 nm and in that spectral region, absorption by haemoglobin and melanin impairs the detection of light originating from deep tissues. For future studies, investigators should consider using other luciferases and luciferin analogues that emit in the near-infrared region[176], [177] for non-invasive imaging that would enable longitudinal studies of lung responses to pulmonary drug delivery.

Photoacoustic imaging (PAI) is a relatively new imaging modality related to optical imaging. In PAI, molecules are excited by laser pulses, and their de-excitation by heat emission generates pressure waves in their environment. These pressure waves can then be detected by ultrasound transducers.[178] Jung *et al.* have recently reported the use of PAI of show the accumulation of a tumour-targeting peptide labelled with a near-infrared dye in lung tumours, although this was administered intravenously.[179] An interesting feature of PAI is its ability to perform label-free imaging, exploiting the optical absorption properties of endogenous molecules such as oxy- and deoxyhaemoglobin, melanin and lipids. Raes *et al.* have used this to show the oxygenation status of orthotopic lung tumours in mice[180] and thus one could envisage using PAI to monitor tumour response to inhaled anti-cancer drugs. To our knowledge there are no reports of photoacoustic imaging of drug delivery by inhalation to date, but the ability of this technique to provide high resolution *in vivo* imaging of deep tissues in small animals would certainly make it a valuable tool for this application.

### f Mass spectrometry imaging

**Fig. 6:**
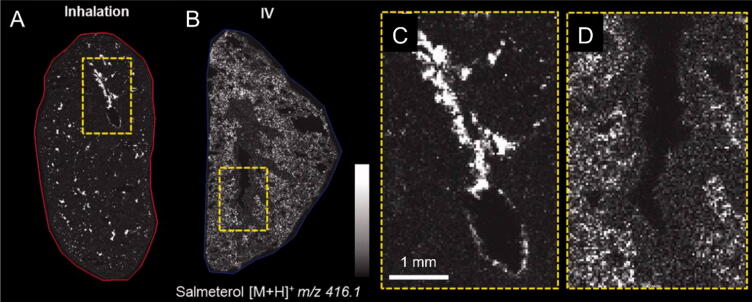
Mass spectrometry imaging of the lungs. MALDI-MS images of 10 μm sections of rat lungs 30 min after administration of salmeterol administered by inhalation (A,C) or intravenous injection (B,D), showing the difference in distribution uniformity between the two administration routes. Adapted from Bäckström et al. [183]

Mass spectrometry (MS) imaging is a molecular imaging technique that is increasingly growing in popularity. The technique is based on the same principles as mass spectrometry but using tissue sections as samples, ionising (typically by laser ablation) successive areas of the sample in raster fashion to determine their composition and map their molecular composition. By determining the precise molecular mass of the collected ions, the identity and concentration of multiple molecules at any given site can be determined with a high degree of confidence and high sensitivity. Molecules of interest can be isotopically labelled (typically with stable isotopes such as deuterium, ^13^C, ^15^N), or imaged label-free based on mass fragmentation patterns. This represents a considerable advantage over imaging techniques that require labelling, since the distribution of an unmodified drug as well as its metabolites can be visualised selectively. Various sampling and ionisation methods exist, such as matrix-assisted laser desorption ionisation (MALDI), secondary ion mass spectrometry (SIMS), laser ablation-inductively coupled plasma-MS (LA-ICP-MS) or desorption electrospray ionization (DESI).[181], [182] Because tissue structures can be identified through their molecular composition, it is also possible to determine the type of tissue in which a drug is present. Therefore, although MS imaging can only be performed *ex vivo*, it has the considerable advantages of providing quantitative and high-resolution (lateral resolution: 5–200 μm depending on the technique employed) information on the spatial distribution of drugs. Bäckström *et al*. compared the lung distribution of salmeterol through different routes by simultaneously administering inhaled nebulised salmeterol (MW 415) and intravenous d_3_-salmeterol (MW 418) to rats. The distribution of d_3_-salmeterol after intravenous injection was homogenous whereas inhaled salmeterol accumulated mainly in the epithelial and subepithelial regions of the bronchioles (Fig. 6).[183] The same team expanded this work to the simultaneous administration of salmeterol, salbutamol and fluticasone propionate (and their deuterated homologues), showing that the distribution of salbutamol and salmeterol after inhalation occurred primarily in bronchiolar tissue whereas fluticasone propionate was more extensively distributed, and that all three drugs were retained longer in lung tissue after inhalation compared to intravenous administration.[184]. A deeper and more uniform deposition of tiotropium bromide after aerosol inhalation compared to intranasal instillation has also been demonstrated in guinea pigs,[185] and aerosolised bexarotene appeared to have relatively uniform distribution in mouse lungs.[186] MS imaging of a bronchial biopsy of a COPD patient taking ipratropium also showed that a region of tissue with low ipratropium uptake was in fact a tumour.[187] Recently, MS imaging was employed to show that the deposition of inhaled ciclesonide in the airways of rats was followed by rapid local metabolism, as ciclesonide was only detected in the lungs but its metabolites were detected both in the lungs and other organs.[188] The ability of MSI to detect metabolites in addition to the original drug is a considerable advantage of other imaging modalities. Finally, by imaging both the encapsulated drug and the main constituent (PLGA) of drug-loaded PLGA microparticles, Robinson *et al.* were able to demonstrate the slow release of the drug from the microparticles into the lungs, whereas a micronized formulation of the same drug with lactose was much more rapidly cleared from the lungs.[189] Thus, whilst not as widespread as some other imaging modalities and restricted to *ex vivo* imaging, MSI has immense potential for the study of inhaled drug delivery.

## 5 Applications in oncology

Compared to its relatively widespread use for studying the delivery of drugs to the lungs to treat respiratory diseases, imaging pulmonary drug delivery in oncology is less common. For the most part, this relates specifically to lung cancer, as a major rationale for using the inhalation route is to target the lungs and reduce side effects associated with systemic delivery of anticancer drugs, albeit with the risk of increased pulmonary toxicity.[10] However, it is also possible to use the inhalation route to target tumours in other organs, and in this section we occasionally highlight such studies. In 2020, lung cancer was by a wide margin the leading cause (18 %) of cancer-related deaths worldwide, and had the highest proportion of new cases in men (14.3 %) and the third-highest incidence (8.4 %) in women after breast cancer and colorectal cancer in women.[190] Many chemotherapeutics and non-cancer drugs have been repurposed for inhalation to treat lung cancer, and for more general coverage of this topic we direct the reader to a detailed review by Lee *et al.*[191].

### a Imaging inhaled small molecules in oncology

**Fig. 7:**
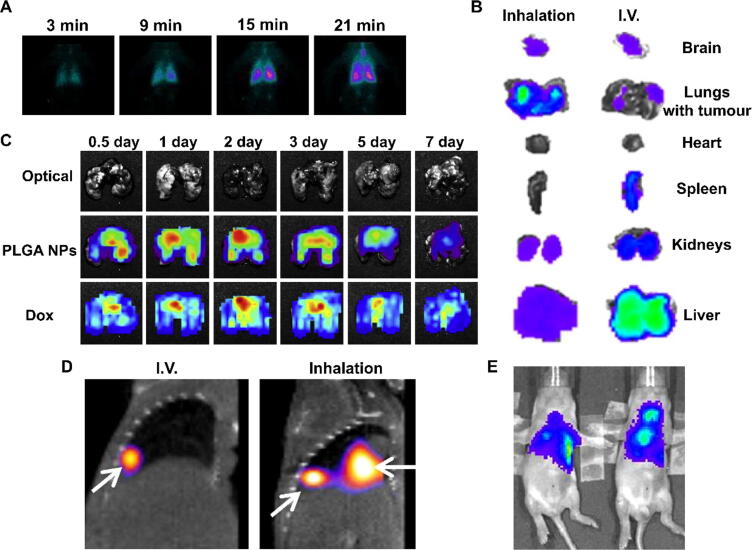
Imaging the pulmonary delivery of nanoparticles, antibodies and cells in lung cancer models. (A) Dynamic scintigraphic images of healthy non-human primates showing the progressive accumulation of ^99m^Tc in the lungs during continuous nebulised delivery of a solution of gemcitabine and [^99m^Tc]Tc-DTPA. Modified with permission from Gagnadoux et al.[194] (B) *Ex vivo* optical imaging showing the distribution patterns of DiR-labelled, paclitaxel-containing nanoparticles after inhalation or intravenous injection in mice bearing lung tumours. Modified from Garbuzenko et al.[226] (C) *Ex vivo* optical imaging of tumour-bearing mouse lungs after pulmonary delivery of PLGA nanoparticles containing both Cy5.5-TRAIL and doxorubicin, imaged at different wavelengths. Adapted from Kim et al.[229] (D) Near-infrared fluorescence tomography and CT imaging (sagittal section) of a mouse bearing a lung tumour, after administration of NIR-labelled cetuximab. Images were taken 13 days after intravenous administration or 48 h after orotracheal administration of the aerosolized antibody. Adapted from Guilleminault et al.[237] (E) Bioluminescence imaging of rats administered luciferase-expressing mesenchymal stem cells intratracheally, showing engraftment of MSCs in the lung and trachea regions. Adapted with permission from Chen et al.[247]

To our knowledge, the only reports of inhaled small-molecule anti-cancer therapy in which *in vivo* imaging was employed are the preclinical and clinical studies of aerosolised gemcitabine labelled with ^99m^Tc.[17], [192], [193], [194] Gagnadoux *et al*. used [^99m^Tc]Tc-DTPA to image the deposition of aerosolised gemcitabine in baboons (Fig. 7A).[194] *In vitro* data showed no difference in tumour-killing efficiency between nebulized and non-nebulized gemcitabine, and no apparent toxicity was observed in the baboons after 9 weeks of weekly gemcitabine inhalation. Dynamic scintigraphic imaging of [^99m^Tc]Tc-DTPA-gemcitabine showed that the target dose was achieved after 21 min of inhalation. In a subsequent clinical study by the same team, scintigraphy revealed that about half of the nominal dose was deposited in the patient’s lungs, with distribution throughout the non-obstructed areas of the lungs although some hotspots were visible.[17] In that study, Lemarié *et al*. also provide an interesting photograph of a specially designed cabinet equipped with air filters in which the patient can inhale the drug without endangering healthcare staff.[17] Indeed, for inhaled cytotoxic chemotherapeutic drugs, air contamination becomes an additional risk for anyone else present during administration, but newer nebulisers that only emit aerosol during the inhalation cycle of patient breathing and good containment practices could help mitigate this risk.

In terms of *ex vivo* imaging, Zhang *et al.* have used mass spectrometry imaging to show the uniform deposition of aerosolised bexarotene in mouse lungs,[186] and the distribution of a fluorescently labelled a microRNA targeting K-Ras-driven tumours in an orthotopic mouse model of lung cancer.[195] In the latter study, the initial deposition of the fluorescent miRNA was high and exclusively in the lungs but rapidly declined, with only residual fluorescence visible in the lungs 24 h after inhalation. This was matched by results from quantitative RT-PCR analysis, suggesting the entire miRNA was cleared or degraded rather than just the fluorescent moiety. In line with its rapid elimination profile, the miRNA was administered twice weekly over 3 weeks to achieve therapeutic efficacy.

Given the small number of published imaging studies to draw lessons from, we have instead attempted to illustrate some potential benefits of incorporating imaging into studies of inhaled anti-cancer chemotherapy. For example, a valuable set of studies showing the superior efficacy of inhaled topotecan over intravenous administration in several orthotopic and heterotopic xenograft models of human lung cancer in rats and mice was recently published.[72], [196] One of the highlights of this work is the efficacy of inhaled topotecan towards tumours that were implanted subcutaneously in the abdomen, demonstrating the utility of the pulmonary route for delivering drugs to extrapulmonary locations. However, the pharmacokinetic analysis was obtained by HPLC analysis of excised tissues, requiring the serial sacrificing of animals and therefore considerably increasing the number of animals used in the study. In the clinic, options are more limited and blood sampling was used as a surrogate measure of drug delivery in a trial of inhaled azacytidine.[20] *In vivo* imaging can provide non-invasive and repeated measurements of drug concentrations and therefore fewer animals are necessary for preclinical studies and more representative measurements can be taken in patients. In the specific case of topotecan, this molecule can be imaged by PET directly as it can be radiolabelled with ^11^C without altering its chemical structure.[197], [198] For azacytidine and gemcitabine, radiolabelled nucleoside analogues such as [^18^F]FIAC[199] or [^18^F]FAC[200] may be useful in evaluating inhaled delivery of these anti-cancer drugs. Imaging either with radiolabelled topotecan or by spiking the aerosol with a different radiotracer could also reveal whether the delivery is affected by the presence of tumour nodules in the lungs. This is currently a less-well studied aspect of inhaled delivery for lung cancers for which significant interindividual variability might be expected. Therapeutic efficacy in the orthotopic models was also evaluated by measuring tumour burden at the end of the study, i.e. by weighing tumour-bearing lungs and subtracting the average weight of age-matched healthy lungs.[72], [196] The growth of tumours in the lungs is indeed difficult to track and while the authors point towards previous validation studies, this approach could be complemented by tumour imaging for more insight at intermediate time points. For example, engineering the tumour cell lines to express an imaging reporter gene such as firefly luciferase would allow the tracking of tumours by BLI and provide real-time data on tumour growth and response to treatment, as shown in a study of inhaled cisplatin in an orthotopic mouse model of lung carcinoma.[201] Alternatively, therapeutic responses can be imaged early with tumour antigen-specific probes, even before tumour volume changes are detectable. For example, by administering a fluorescent probe targeting integrin ανβ3 to mice bearing orthotopic syngeneic lung tumours, significant differences in integrin ανβ3 expression in response to treatment were apparent one week after first tumour growth, whereas changes in tumour volume were only apparent after 2 weeks.[202] Although this study did not involve inhaled delivery, it demonstrates the value of *in vivo* optical imaging for longitudinal evaluation of treatment response in preclinical models of lung cancer, especially combined with CT,[158] and this approach should be equally usable after pulmonary drug delivery. Another study that unveiled the relationship between imaging signal and drug resistance biomarker expression indicated that the clearance rate of inhaled [^99m^Tc]Tc-sestamibi was correlated to the expression level of multidrug resistance protein 1 (MRP1) in healthy lung tissue, with a much slower clearance rate in patients with high expression of MRP1.[203] Earlier detection of response to treatment could lead to quicker clinical decisions on whether to pursue a certain course of treatment in a patient.

### b Imaging inhaled nanomedicines in oncology

There are more reports of drug delivery imaging of inhaled nanomedicines in oncology compared to small molecules, presumably because they are easier to functionalise for imaging purposes. Here we focus on recent examples of inhaled nanomaterials – defined as particulate matter < 1 μm in diameter – and biologics, in particular antibody-based therapeutics and cell therapies. Imaging of inhaled nanoparticles in oncology is important from two different perspectives. First, nanoparticles can be used both as vehicles for drug delivery and as imaging agents. The versatility and diversity of nanoparticles in terms of materials, size, surface charge, loading capacity and surface modifications has made them attractive and promising targets for drug development. Second, inhaled nanoparticles and in particular environmental nanoparticles, e.g. from vehicle exhaust gases, industrial sources, building materials, etc, are known to contribute to numerous diseases such as cancer, airway inflammation, and fibrosis. For a general review on the preparation and use of radiolabelled nanoparticles in medicine, we refer the readers to a recent review.[204] Particles smaller than 100 nm are deposited in the lungs by diffusion only, whereas particles of diameters in the range of 100 nm to 1 μm are deposited both by diffusion and sedimentation.[205] After deposition, many parameters including size, material, surface charge and coating affect the pharmacokinetics of nanoparticles such as their clearance and translocation pathways.[206].

In the case of liposomes, it has long been established that their delivery by inhalation leads to long retention in the lungs. Although no imaging was performed, early studies of liposomes encapsulating tritiated cytosine arabinoside A ([^3^H]Ara-C) demonstrated that intratracheal delivery resulted in longer retention of [^3^H]Ara-C in the lungs and much lower plasma levels of [^3^H]Ara-C metabolites than the non-encapsulated drug, suggesting the use of inhaled liposomes to reduce side effects of chemotherapy.[207] A notable example involving imaging is the clinical study by Farr *et al.*,[208] in which ^99m^Tc-labelled liposomes were administered to healthy volunteers using a jet nebuliser. This study showed the influence of the initial deposition on the fate of the liposome, as deposition in the higher airways lead to much faster clearance from the lungs compared to those that deposit more peripherally, beyond the mucociliary escalator.

Wu *et al*. evaluated the delivery of gadolinium-labelled perfluorocarbon (PFC) nanoparticles in a rabbit model of lung cancer using ^1^H and ^19^F MRI.[209] ^19^F is an attractive nucleus to use for MRI as it has no background signal in the body, and imaging characteristics similar to ^1^H. PFCs have also been studied extensively as potential blood substitutes and ultrasound imaging agents (as microbubbles) and are therefore well characterised biologically.[123] Nanoparticles administered intratracheally were found to persist in the lungs for at least 72 h, whereas after intravenous administration there was only transient accumulation in the lungs in the first hours after injection. Furthermore, the structure of these nanoparticles, with ^19^F present in the PFC core and gadolinium localised on the outer shell, provided an elegant way to assess the stability of the construct: in this case the colocalization of ^1^H and ^19^F MRI signals suggested these particles were stable within the tumour environment. To evaluate drug delivery, the PFC nanoparticles were additionally labelled with a fluorescent dye, which revealed after microscopic examination that intratracheal administration resulted in penetration of the dye deep into the tumour tissue, which was not observed after intravenous administration. The long persistence of PFC nanoparticles and absence of toxicity in the lungs could be a significant advantage for drug delivery in lung cancer, as other types of nanoparticles (SPIO, TiO_2_) have been found to cause local inflammation and/or to enter the circulation after inhalation and cause toxicity in the kidneys.[210], [211] Also using MRI, Howell *et al*. prepared lipid-micellar nanoparticles containing a manganese oxide core (for MRI) to deliver DNA and doxorubicin.[212] DNA was bound by electrostatic interactions to cationic lipid micelles. Intranasal administration was used to deliver particles and doxorubicin to the lungs of mice, with limited distribution in other organs, whereas the intravenous route resulted in mostly liver, kidney and spleen uptake. Bianchi *et al*. conducted a series of studies using ultrasmall (<5 nm) polysiloxane nanoparticles labelled with gadolinium on their surface and additionally conjugated to a near-infrared dye, imaging them by UTE-MRI. Whilst in healthy rats the intratracheally administered nanoparticles showed relatively rapid and uniform distribution in the lungs followed by translocation into the bloodstream and renal elimination (Fig. 5D),[135], [213] in rats bearing lung tumours the accumulation of the nanoparticles was much more localised to the tumoured areas and their retention was prolonged.[214] The purported reason for this accumulation was the absence of a functional alveolar barrier in the tumour areas. There was a good correlation between the BLI signal intensity from the luciferin-expressing tumours and the tumour volume determined from the MR images, therefore these particles could be useful to improve tumour delineation by MRI. Interestingly, these particles were also observed to accumulate in brain tumours after both orotracheal and intravenous delivery.[215] These particles have since undergone clinical trials as MR-imageable radiosensitisers, albeit by intravenous administration, with encouraging safety and efficacy data.[216], [217]

Inhaled nanoparticles have also been evaluated as potential agents for lymphoscintigraphy. Solid lipid nanoparticles were labelled with the lipophilic radiotracer ^99m^Tc-HMPAO and administered as aerosols into healthy rats, showing rapid and extensive uptake in lymph nodes and very little uptake in the liver.[218], [219] This suggests little passage of these nanoparticles into the circulation and could represent an alternative method to perform lymphoscintigraphy with much lower background signal in the subject.

A number of studies have been conducted with fluorescently labelled nanoparticles in small animal models of cancer, frequently aimed at delivering nucleic acids.[220], [221], [222] For example, LHRH-targeted and non-targeted lipid nanoparticles carrying anti-tumoural siRNAs were fluorescently labelled and their distribution compared after inhalation in mice bearing lung tumours. Although imaging was only performed *ex vivo*, it revealed that the targeted nanoparticles preferentially accumulated in tumour-affected regions of the lungs, whereas the non-targeted particles were more evenly distributed throughout the lungs.[223] Exosomes, having on their surface numerous molecules that can mediate interactions with tumour cells, were also observed to preferentially target lung metastases after inhalation.[224] Another study aimed at comparing several types of nanoparticles (e.g. liposomes, micelles, quantum dots, PEG polymers, etc) found that most nanoparticles had higher accumulation and longer persistence in the lungs after inhalation than after intravenous administration.[225] By exploiting fluorescence quenching of labelled siRNA and paclitaxel packed together in lipid nanoparticle, the same authors were able to image the release of the drugs from the nanoparticles after cellular internalisation *in vitro* (Fig. 7B).[226] Another possibility is to use “smart” or “responsive” systems that release drugs upon cleavage by a tumour-associated enzyme. For example, Wang *et al*. generated paclitaxel-loaded matrix metalloproteinase (MMP)-reactive micelles by using succinylated gelatine, which is a substrate for MMP2/9, as a stabiliser. The presence of MMP2/9 in the tumour microenvironment leads to gelatine degradation, releasing paclitaxel in the surrounding tissue. Whilst in this study only the deposition and retention of the micelles in the lungs of rats was assessed by *ex vivo* NIR fluorescence imaging,[227] a drug delivery imaging system based on the same principle is easily conceivable. Loading an activatable probe that fluoresces upon cleavage by MMP would reveal the release of the drug at the tumour site and not just the presence of the drug carrier.

Larger, micron-sized particles have also been used in bids to reduce uptake by macrophages. Using the intrinsic fluorescence of doxorubicin (470/560 nm), Kim *et al*. prepared doxorubicin-loaded porous PLGA microparticles and administered them intratracheally with a dry powder insufflator to mice bearing lung metastatic melanomas.[228] By *ex vivo* imaging, doxorubicin was observed to progressively distribute throughout the lungs from an initially central deposition area, over the course of 2 weeks. This was attributed to the slow release of doxorubicin from the porous microparticles. When the particles were surface-coated with TRAIL for additional tumour targeting, the fluorescently labelled TRAIL was released from the particles more rapidly than the more deeply embedded doxorubicin, both *in vitro* and *in vivo* (Fig. 7C)*.*[229] Also using *ex vivo* fluorescence imaging of tumour-bearing mouse lungs, Rosière *et al*. studied lipid micelles and chitosan-based liposomes for paclitaxel delivery by inhalation, and the diffuse pattern of fluorescence demonstrated penetration of these nanoparticles deep into the lung tumour independently of local tumour vascularisation.[230], [231]

The airway mucus is a significant barrier for inhaled drugs, and numerous strategies to overcome this barrier have been devised. Amongst them, low-MW PEGylation appears to be effective, as evidenced by images of PEG2k-ferritin nanocages penetrating through the mucus of mouse airways, in clear contrast to non-PEGylated nanocages that appeared to aggregate within the airways and nanocages PEGylated with longer PEG chains (5–10 kDa) that simply coated the airways without penetrating further.[232] A common point to all these studies is the use of fluorescent dyes that have little tissue penetration and thus prevent *in vivo* imaging. Despite using a fluorophore with a 670 nm emission peak, Capel *et al.* were unable to detect a signal by *in vivo* imaging of inhaled chitosan-siRNA complexes, whereas *ex vivo* imaging revealed very heterogenous delivery in the lungs.[233] Deep-seated tissues such as the lungs are more challenging to imaging non-invasively by fluorescence because of light absorption and scatter by surrounding tissues, particularly the ribcage. In addition, chlorophyll fluoresces in the 700 nm region and can lead to relatively high background signal. This can be problematic for lung imaging because of the physical proximity of the lungs and the GI tract, as shown by Zhou *et al*.[234] To avoid this issue, animals can be fed a chlorophyll-free diet for a week prior to imaging, but surprisingly this is not frequently reported in studies. Whilst *ex vivo* fluorescence imaging is useful to confirm drug delivery *a posteriori*, a different selection of fluorescent dyes more amenable to *in vivo* imaging might have allowed to visualise drug distribution longitudinally after lung delivery, potentially demonstrating the different fates of drugs and carriers over time. For example, cetuximab labelled with Xenofluor 750^TM^ was visible in the lungs for up to 72 h by *in vivo* reflectance (i.e. 2D) fluorescence imaging after inhalation, but not after intravenous administration despite *ex vivo* imaging demonstrating its presence.[235] Whilst longitudinal imaging provided qualitative assessment of antibody distribution, 2D fluorescence imaging lacks sensitivity and quantification. More recent instruments capable of tomographic imaging have allowed 3-dimensional visualisation of fluorescently labelled nanoparticles[236] and cetuximab (Fig. 7D).[237] The latter study in particular provided image-derived quantification of antibody levels in the lungs for up to 42 days after administration, comparing not only the inhalation route to the intravenous route but also examining the role of the FcRn receptor in the fate of antibodies and suggesting the presence of additional mechanisms to FcRn mediating the transport of antibodies from the lungs to the circulation. The long period over which imaging was possible is a significant advantage of fluorescence imaging, particularly over nuclear imaging. *In vivo* fluorescence imaging is therefore a powerful tool, when optimally used, to evaluate drug delivery in small animal models.

Imaging drug delivery can also be performed with a “pre-targeting” approach, which decouples the administration of the drug moiety from the administration of an imaging moiety for the biological target. Binding of the two moieties happens *in vivo*, through selective interactions such as the streptavidin–biotin pair or bioorthogonal conjugation reactions, for example”click” reactions such as strain-promoted azide-alkyne cycloadditions (SPAAC) or inverse electron demand Diels-Alder (IEDDA) reactions. This is beneficial when the binding of the drug to its target is affected by the presence of a bulky imaging moiety, when the pharmacokinetics of the drug of interest are not compatible with the imaging moiety (e.g. a slow-accumulating drug and a short-lived radionuclide), or for theranostic approaches combining an imaging and a therapeutic radionuclide.[238], [239] An example of this approach with pulmonary delivery is the use of an oxidised version of avidin nebulised into mice bearing lung tumours. Biotinylated therapeutic antibodies could then be used to specifically bind the avidin and thereby increase their residence time in the lungs. Intravenous administration of ^64^Cu- and ^111^In-labelled biotin 24 h after avidin inhalation resulted in a 10–30× higher accumulation of activity in the lungs of avidin-treated mice compared to control-treated mice, demonstrating the presence and retention of the oxidised avidin.[240].

Despite the increasing number of antibodies (and antibody fragments or derivatives) approved as therapeutics, only two inhaled protein-based drug has received regulatory approval to date (Afrezza®, an inhaled formulation of insulin, and Pulmozyme®, a recombinant human deoxyribonuclease for CF treatment). Amongst the reasons for this are the high production cost of these drugs coupled with the low efficacy of the inhalation route, and protein instability during aerosolization.[241], [242] Additionally, the rapid clearance of inhaled antibodies from the airways [243] is an obstacle to the use of this route for antibody delivery, whereas intravenously administered antibodies have long circulation times (in the case of IgG-class antibodies, which all currently approved mAbs belong to) and therefore better potential for long-term therapeutic efficacy. A comparison of inhaled IgG, IgA and IgAM showed that this rapid elimination appears to be relatively independent of antibody class.[244] Attempting to circumvent this rapid clearance, Koussoroplis *et al.* conjugated antibody fragments to 40-kDa PEG chains and found that this procedure led to an increased retention of antibodies in the lungs, primarily through increased mucoadhesion and reduced uptake by macrophages.[245] IgE-class antibodies, which are known to have long tissue residence times despite extremely rapid clearance from the circulation and are currently investigated as anti-cancer therapeutics,[246] might also be interesting candidates for pulmonary delivery. In any case, imaging has considerable potential to assist the development of inhaled antibodies by providing quantitative, real-time biodistribution information.

Finally, the recent progresses in cell therapies have also brought forward the issue of delivering therapeutic cells to the lungs.[248] In particular, mesenchymal stem cells (MSCs) have the potential to be used for regenerative therapy in lung diseases [249], [250] and as anti-cancer agents.[251], [252] Cell therapies are typically delivered intravenously, which results in transient trapping in the pulmonary vasculature followed by the accumulation of a large fraction of administered cells in the liver and spleen [253] despite the abilities of MSCs to home to sites of disease. The homing of MSCs to lung tumours and metastases after intravenous administration has been imaged by MRI,[254] BLI (Fig. 7E) [247] and PET,[255] in the latter case showing viable MSCs present in the lungs up to 1 week after administration. Delivering MSCs directly to the lungs may increase the efficacy of such therapies and reduce the risk of intravascular cell aggregation, but studies of the topic are still in their infancy.[247], [256] Recent studies have shown that pulmonary delivery of cells by aerosolization is feasible with careful device selection,[257], [258], [259], [260] as well as intranasal delivery.[261] Considering the very high production costs of cell therapies,[262] it is important to choose an efficient delivery method to reduce wastage,[263] and non-invasive imaging will be useful in determining the optimal delivery method. Amongst the few studies incorporating imaging of therapeutic cells after pulmonary delivery, a landmark study showed the transfer of mitochondria from intranasally instilled MSCs to the alveolar epithelium by fluorescence microscopy in *ex vivo* perfused mouse lungs, resulting in protection against acute lung injury.[264] Another notable example is a study by Kim *et al.,* in which fluorescently labelled MSCs were administered into the trachea of rats by means of a liquid gelatine plug and imaged with a bronchoscopic probe as well as a transpleural probe, revealing the deposition of MSCs in the alveoli.[265] Further studies should attempt to establish whether the precise localisation or number of administered cells correlate with therapeutic efficacy. To study the long-term engraftment of therapeutic cells in the lungs after pulmonary delivery, reporter gene imaging will be a useful approach.[266]

## 6 Conclusions and perspectives

Despite its apparent simplicity, the imaging studies reviewed here have shown that efficient drug delivery to the lung via inhalation is a rather complex process that starts from the design and composition of the drug delivery systems to the administration devices used. For example, it is clear that the different techniques to deliver the drug to the lungs suffer from several important technical challenges to overcome (e.g. contamination of the environment with aerosol, efficient lung deposition) that other established administration routes such as intravenous do not have. In addition, the lung presents several biological barriers that are specific to this organ, and hence require dedicated solutions and design elements that have not been fully developed and may or not align with those that have been traditionally shown effective for drug delivery systems designed for other administration routes.

Overcoming the barriers to inhaled drug delivery requires understanding them better. In this review we have described the main imaging modalities applicable to inhaled drug delivery. There is now a large array of tools available, i.e. instrumentation and tracers, to elucidate the fate of inhaled drugs, at scales ranging from a whole patient to the cellular or subcellular level. We encourage those working in inhaled drug delivery to reach out to the imaging community and make use of these solutions, as the appropriate imaging method will help uncover the underlying issues they might face. For example, an inhaled anti-cancer drug formulation may lack efficacy in clinical trials, and nuclear imaging with a radiolabelled drug would be able to demonstrate that this is due to the formulation failing to reach the affected parts of the lung for structural or aerodynamic reasons, or it might reach the area rapidly, but the drug may not be retained long enough to have an effect. Alternatively, optical or mass spectrometry imaging in preclinical models may reveal that the drug, despite generally high deposition and retention in the lungs, is actually phagocyted by lung macrophages, or subject to rapid intracellular metabolism. Thus, optimal use of imaging methods could solve issues in the drug development process and, on a more fundamental level, improve our understanding of the various physiological and biochemical processes at work inside the lungs.

This is illustrated by many of the imaging studies discussed here, which highlight several important findings that provide a more positive outlook for this field. For example, it seems clear that suitably designed or formulated drugs administered via inhalation show a better lung retention profile than with intravenous administration. This should encourage efforts into developing inhaled formulations of both current and future anti-cancer drugs in order to maximise their efficacy and improve their safety profile.

Additionally, in terms of design principles, nano-sized particles provide opportunities to optimise intra-lung distribution after deposition, and that aerosol deposition in the peripheral airways can avoid rapid mucociliary clearance from the lungs. Importantly, it appears that molecularly targeted nanoparticles do indeed show preferential tumour uptake compared to non-targeted probes that distribute more homogeneously throughout the lung. This is unlike intravenously-injected nanoparticle drug delivery systems that mostly rely on the enhanced penetration and retention (EPR) effect for effective accumulation in the tumour, regardless of being molecularly targeted or not. Other modifications of the surface of nanoparticles also appear to be beneficial in helping drugs and formulations penetrate lung mucus, resulting in higher payload delivery to the target tissue. Overall, these imaging-derived findings appear informative for the future design of future inhalation-based lung cancer drug delivery systems and importantly, would have been challenging to obtain without the use of imaging methods.

It is likely that the lack of progress in the clinical translation of these systems to date has been affected by some of the issues discussed above, and we hope that the imaging studies identified and discussed in this review provide useful information that, taken together, will support the design and development of future drug delivery systems and the developments in imaging technology and methods will facilitate their progress through pre-clinical and clinical phases of medicines development.

## Declaration of Competing Interest

The authors declare that they have no known competing financial interests or personal relationships that could have appeared to influence the work reported in this paper.
